# Supplementary material for: The Soybean Laccase Gene Family: Evolution and Possible Roles in Plant Defense and Stem Strength Selection
Source: Genes (Basel). 2019 Sep 11;10(9):701. doi: 10.3390/genes10090701 (PMC6770974; doi:10.3390/genes10090701)
Supplement: Supplementary file 1 [file genes-10-00701-s001.pdf]

Table S1. Basic information of soybean laccases. (Abbreviations and columns requiring detailed explanation are marked with an asterisk and explanations are given at the bottom of the table)

Gene name	Gene ID	Chr*	Physical location	AAL(aa)*	MW(D)*	pI*	SL*	HRL(aa)*	TD*	SP*	PREDICTION*
GmLac1	*Glyma.01G108200*	Chr1	36947944-36957036	567	62552.64	5.58	Extracellular	14	0	Y	n4-18c23/24o
GmLac2	*Glyma.01G112600*	Chr1	38353594-38358163	557	61144.5	9.14	Extracellular	8	0	Y	n8-16c22/23o
GmLac3	*Glyma.01G173500*	Chr1	51045827-51048552	561	61859.84	8.08	Extracellular;Lysosomal	11	0	Y	n9-20c27/28o
GmLac4	*Glyma.01G173600*	Chr1	51051683-51054726	564	62548.75	8.85	Lysosomal	11	0	Y	n13-24c30/31o
GmLac5	*Glyma.01G183100*	Chr1	51843424-51847358	540	60379.43	8.77	Lysosomal	11	0	Y	n6-17c24/25o
GmLac6	*Glyma.02G224800*	Chr2	41222747-41227060	557	61357.92	9.41	Extracellular	11	0	Y	n8-19c23/24o
GmLac7	*Glyma.02G231600*	Chr2	41882853-41885826	575	63054.23	8.98	Extracellular;PlasmaMembrane	11	0	Y	n11-22c29/30o
GmLac8	*Glyma.02G261600*	Chr2	44758969-44763198	569	62620.24	6.97	Extracellular	11	0	Y	n8-19c24/25o
GmLac9	*Glyma.03G077900*	Chr3	19361933-19366381	557	61150.55	9.1	Extracellular	7	0	Y	n9-16c22/23o
GmLac10	*Glyma.04G019500*	Chr4	1525860-1533999	547	60677.86	9.42	Extracellular	18	0	Y	n5-23c28/29o
GmLac11	*Glyma.04G119600*	Chr4	14443110-14447983	592	65989.74	6.9	Extracellular;PlasmaMembrane	13	1	Y	n4-17c23/24o569-591i
GmLac12	*Glyma.05G056100*	Chr5	5123859-5128473	597	67145.88	9.09	Extracellular	15	0	Y	n9-24c29/30o
GmLac13	*Glyma.05G082700*	Chr5	13157352-13160970	425	47723.8	9.35	Extracellular		0	0	i
GmLac14	*Glyma.06G019800*	Chr6	1494230-1501925	547	60813.04	9.38	Extracellular	18	0	Y	n5-23c28/29o
GmLac15	*Glyma.06G284300*	Chr6	47271762-47274605	396	43729.76	8.91	Extracellular;PlasmaMembrane	7	1	Y	n2-9c13/14o37-57i
GmLac16	*Glyma.06G307100*	Chr6	49594181-49599176	537	60120.76	7.63	Extracellular	10	0	Y	n7-17c22/23o
GmLac17	*Glyma.06G318800*	Chr6	50742178-50747062	591	65687.37	6.47	Extracellular;PlasmaMembrane	14	1	Y	n4-18c23/24o569-590i
GmLac18	*Glyma.07G054100*	Chr7	4733502-4736103	572	64035.46	7.63	Extracellular;PlasmaMembrane	11	0	Y	n4-15c20/21o
GmLac19	*Glyma.07G054200*	Chr7	4742597-4747000	572	64096.48	8.72	Extracellular;PlasmaMembrane;Lysosomal	8	0	Y	n4-12c20/21o
GmLac20	*Glyma.07G133900*	Chr7	15868910-15873228	579	64138.44	9.22	Extracellular;PlasmaMembrane;Lysosomal	20	1	0	i12-32o
GmLac21	*Glyma.07G134100*	Chr7	15880168-15883379	584	65179.59	9.94	Mitochondrial;Peroxisomal	11	0	Y	n14-25c31/32o
GmLac22	*Glyma.07G142400*	Chr7	16921847-16926142	572	63025.16	8.66	Extracellular	11	0	Y	n3-14c22/23o
GmLac23	*Glyma.07G142500*	Chr7	16929178-16933812	573	63888.18	8.79	Extracellular	15	0	Y	n3-18c24/25o
GmLac24	*Glyma.07G142600*	Chr7	16975838-16981175	570	63930.58	8.71	Extracellular;PlasmaMembrane	11	0	Y	n5-16c21/22o

GmLac25	*Glyma.07G225300*	Chr7	40239363-40241517	550	61539.15	9.08	Extracellular;Lysosomal	11	0	Y	n4-15c22/23o
GmLac26	*Glyma.07G225400*	Chr7	40248750-40251212	552	61888.46	8.96	Extracellular;Lysosomal	11	0	Y	n4-15c23/24o
GmLac27	*Glyma.07G261800*	Chr7	43671355-43676147	547	61959.43	9.55	Extracellular;Lysosomal	12	0	Y	n10-22c27/28o
GmLac28	*Glyma.08G138900*	Chr8	10636329-10638034	448	50654.41	8.17	Extracellular	18	1	0	o12-30i
GmLac29	*Glyma.08G343500*	Chr8	45872298-45879050	637	71749.27	6.19	PlasmaMembrane	11	2	Y	n7-18c23/24o47-65i612-635o
GmLac30	*Glyma.08G353500*	Chr8	46665729-46668560	581	64826.14	9.93	PlasmaMembrane	10	0	Y	n11-21c28/29o
GmLac31	*Glyma.08G359100*	Chr8	47105388-47108727	608	67156.99	8.72	Extracellular;PlasmaMembrane;Lysosomal		1	0	i37-58o
GmLac32	*Glyma.08G359200*	Chr8	47112555-47115278	519	57156.63	8.95	Extracellular	11	0	Y	n10-21c26/27o
GmLac33	*Glyma.08G359300*	Chr8	47117261-47120794	578	64121.58	9.07	Extracellular;PlasmaMembrane	11	0	Y	n10-21c26/27o
GmLac34	*Glyma.08G359400*	Chr8	47124368-47126962	440	48810.27	9.14	PlasmaMembrane	7	0	Y	n2-9c15/16o
GmLac35	*Glyma.09G132400*	Chr9	33011543-33014363	523	58489.42	5.2	Extracellular		0	0	i
GmLac36	*Glyma.10G197300*	Chr10	42857995-42860990	600	66239.56	5.2	Extracellular	12	0	Y	n12-24c29/30o
GmLac37	*Glyma.10G219100*	Chr10	45096674-45100122	564	63296.26	8.62	Extracellular;PlasmaMembrane	11	0	Y	n7-18c26/27o
GmLac38	*Glyma.10G219200*	Chr10	45102350-45106419	565	63070.66	6.34	Extracellular;PlasmaMembrane;Lysosomal	13	0	Y	n9-22c26/27o
GmLac39	*Glyma.10G263800*	Chr10	48805999-48811533	575	65408.84	8.87	Mitochondrial;Peroxisomal	11	0	Y	n6-17c22/23o
GmLac40	*Glyma.11G059200*	Chr11	4474609-4478620	537	60246.29	8.88	Lysosomal	11	0	Y	n6-17c21/22o
GmLac41	*Glyma.11G069500*	Chr11	5231970-5235047	564	62462.59	8.76	Extracellular;Lysosomal	11	0	Y	n13-24c30/31o
GmLac42	*Glyma.11G069600*	Chr11	5237575-5240089	562	62447.57	8.5	Extracellular;PlasmaMembrane;Lysosomal	11	0	Y	n10-21c28/29o
GmLac43	*Glyma.11G097300*	Chr11	7426344-7431229	547	61503.03	9.36	Extracellular;Lysosomal	11	0	Y	n13-24c31/32o
GmLac44	*Glyma.11G137500*	Chr11	10456742-10461383	586	65101.49	8.87	Extracellular;PlasmaMembrane;Lysosomal	12	0	Y	n11-23c31/32o
GmLac45	*Glyma.11G164000*	Chr11	15441595-15444592	573	63472.9	9.11	PlasmaMembrane	11	0	Y	n10-21c29/30o
GmLac46	*Glyma.11G233400*	Chr11	32873226-32877927	587	64754.13	9.24	Extracellular	11	0	Y	n16-27c32/33o
GmLac47	*Glyma.11G236800*	Chr11	33157016-33161441	568	63124.69	5.96	PlasmaMembrane;Lysosomal	11	0	Y	n8-19c23/24o
GmLac48	*Glyma.11G256700*	Chr11	34639433-34641547	552	62111.94	8.97	Lysosomal	12	0	Y	n6-18c23/24o
GmLac49	*Glyma.12G023300*	Chr12	1711347-1715917	544	61051.54	9.46	Extracellular;Lysosomal	11	0	Y	n12-23c28/29o

GmLac50	*Glyma.12G060900*	Chr12	4423650-4427688	590	65611.01	9.02	Extracellular;PlasmaMembrane;Lysosomal	11	0	Y	n12-23c31/32o
GmLac51	*Glyma.12G097700*	Chr12	8390539-8395322	537	60204.81	6.83	Extracellular	11	0	Y	n8-17c22/23o
GmLac52	*Glyma.12G121700*	Chr12	13091668-13095659	556	61173.8	9.34	Extracellular	11	0	Y	n8-19c23/24o
GmLac53	*Glyma.12G192800*	Chr12	35434955-35439253	536	59726.15	6.86	Extracellular	11	0	Y	n6-17c22/23o
GmLac54	*Glyma.13G033600*	Chr13	10575983-10578863	315	34958.38	5.7	Extracellular;PlasmaMembrane		0	0	i
GmLac55	*Glyma.13G076900*	Chr13	18197033-18205367	576	64375.78	8.69	Extracellular;PlasmaMembrane;Lysosomal	11	0	Y	n8-19c25/26o
GmLac56	*Glyma.13G338100*	Chr13	43086954-43090229	458	50768.09	9.31	Extracellular		0	0	i
GmLac57	*Glyma.14G041300*	Chr14	3116248-3122893	581	65292.49	8.68	Extracellular;Lysosomal	11	0	Y	n13-24c29/30o
GmLac58	*Glyma.14G056100*	Chr14	4452088-4456161	569	62633.15	6.76	Extracellular	12	0	Y	n7-19c24/25o
GmLac59	*Glyma.14G062300*	Chr14	5075491-5079469	554	61347.32	7.68	PlasmaMembrane	11	0	Y	n5-16c21/22o
GmLac60	*Glyma.14G191500*	Chr14	45616466-45620640	557	61305.75	9.45	Extracellular	11	0	Y	n8-19c23/24o
GmLac61	*Glyma.14G198900*	Chr14	46404283-46407292	575	63299.47	9.04	PlasmaMembrane	11	0	Y	n13-24c29/30o
GmLac62	*Glyma.14G223000*	Chr14	48811743-48817333	547	60830.78	9.08	Extracellular	11	0	Y	n9-20c28/29o
GmLac63	*Glyma.15G109000*	Chr15	8563266-8566506	456	52004.49	9.78	Extracellular;PlasmaMembrane;Lysosomal	11	0	Y	n3-14c19/20o
GmLac64	*Glyma.16G158400*	Chr16	31863760-31867327	566	63868.85	7.67	Lysosomal	11	0	Y	n9-20c25/26o
GmLac65	*Glyma.17G012300*	Chr17	950397-955232	549	62064.49	9.46	Extracellular;Lysosomal	11	0	Y	n10-21c29/30o
GmLac66	*Glyma.17G138300*	Chr17	11183728-11188961	592	66723.58	9.28	Extracellular;PlasmaMembrane	13	1	Y	n6-19c24/25o575-591i
GmLac67	*Glyma.17G180400*	Chr17	20487778-20491962	541	60333.25	8.48	Extracellular;Lysosomal	11	0	Y	n6-17c25/26o
GmLac68	*Glyma.17G180500*	Chr17	20576340-20582231	544	60437.9	9.22	Extracellular;PlasmaMembrane;Lysosomal	11	0	Y	n9-20c24/25o
GmLac69	*Glyma.17G261500*	Chr17	41526584-41534998	541	59959.72	8.83	Extracellular	11	0	Y	n5-16c21/22o
GmLac70	*Glyma.18G023600*	Chr18	1723409-1728464	589	64993.37	9.32	Extracellular	13	0	Y	n16-29c34/35o
GmLac71	*Glyma.18G057200*	Chr18	5045036-5048066	573	63432.79	8.94	Extracellular;PlasmaMembrane	11	0	Y	n10-21c29/30o
GmLac72	*Glyma.18G065100*	Chr18	5925631-5930200	556	61316.06	9.41	Extracellular;Lysosomal	11	0	Y	n8-19c23/24o
GmLac73	*Glyma.18G177200*	Chr18	42244982-42247802	526	58231.06	9.5	Extracellular;PlasmaMembrane;Lysosomal	11	0	Y	n10-21c26/27o
GmLac74	*Glyma.18G177300*	Chr18	42252244-42255072	578	63733.22	9.24	Extracellular;Lysosomal	11	0	Y	n10-21c26/27o
GmLac75	*Glyma.18G177400*	Chr18	42324003-42327195	580	63849.25	8.76	Extracellular;Lysosomal	11	0	Y	n9-20c28/29o

GmLac76	*Glyma.18G183500*	Chr18	44265156-44269294	576	64146.38	9.01	Extracellular;PlasmaMembrane;Lysosomal	11	0	Y	n14-25c31/32o
GmLac77	*Glyma.18G183700*	Chr18	44276577-44279879	584	65012.47	9.96	PlasmaMembrane;Mitochondrial;Peroxisomal	11	0	Y	n14-25c31/32o
GmLac78	*Glyma.18G193200*	Chr18	46559987-46565879	544	59793.03	6.33	Extracellular	11	0	Y	n3-14c22/23o
GmLac79	*Glyma.18G193300*	Chr18	46576771-46581234	560	62307.96	7.02	Extracellular	11	0	Y	n3-14c22/23o
GmLac80	*Glyma.18G193400*	Chr18	46622868-46628926	571	64252.92	8.59	Extracellular;Lysosomal	11	0	Y	n5-16c21/22o
GmLac81	*Glyma.18G197400*	Chr18	47370198-47374339	533	58013.23	8.78	Extracellular	7	0	Y	n8-15c21/22o
GmLac82	*Glyma.20G025200*	Chr20	2743092-2745282	557	62652.49	9.14	Extracellular;Lysosomal	11	0	Y	n14-25c32/33o
GmLac83	*Glyma.20G051600*	Chr20	11622159-11626958	442	49441.43	7.33	Extracellular		0	0	i
GmLac84	*Glyma.20G051700*	Chr20	11685747-11690052	574	64191.39	7.13	Lysosomal	11	0	Y	n6-17c22/23o
GmLac85	*Glyma.20G051900*	Chr20	11769051-11777145	575	64133.39	8.45	Extracellular;Lysosomal	11	0	Y	n6-17c22/23o
GmLac86	*Glyma.20G126500*	Chr20	36862931-36869680	575	65117.41	8.77	Mitochondrial;Peroxisomal	11	0	Y	n6-17c22/23o
GmLac87	*Glyma.20G172600*	Chr20	41019363-41023633	566	63205.74	6.41	Extracellular;PlasmaMembrane;Lysosomal	13	0	Y	n9-22c26/27o
GmLac88	*Glyma.20G172700*	Chr20	41025526-41028921	565	63324.14	9.05	Extracellular;PlasmaMembrane;Lysosomal	11	0	Y	n7-18c22/23o
GmLac89	*Glyma.20G192800*	Chr20	43186683-43190491	598	66247.4	4.95	Extracellular	11	0	Y	n13-24c29/30o
GmLac90	*Glyma.20G192900*	Chr20	43192981-43196211	603	66286.9	5.33	Extracellular;Vacuole		1	0	o584-602i/
GmLac91	*Glyma.U027300*	scaffold_27	174393-178911	600	67074.41	6.79	Extracellular	11	0	Y	n30-41c49/50o
GmLac92	*Glyma.U027400*	scaffold_27	180254-185442	571	63410.1	6.51	Extracellular	14	0	Y	n4-18c23/24o
GmLac93	*Glyma.U040600*	scaffold_614	3780-6730	577	65273.03	7.34	Extracellular	15	0	Y	n10-25c33/34o

Note: **Chr** (Chromosome); **AAL** (Amino acids length); **MW** (Molecular weight); **SL** (Subcellular localization); **HRL** (Hydrophobic region Length); **TD** (Transmembrane domain): The numbers indicate how many predicted transmembrane segments; **SP** (Signal peptide): Y/N indicat if a signal peptide was predicted or not; **PREDICTION**: Predicted topology of the protein. The topology is given as the position of the transmembrane helices separated by "i" if the loop is on the cytoplasmic or "o"if it is on the non cytoplasmic side. A signal peptide is given by the position of its h-region separated by a "n" and a "c", and and the position of the last amino acid in the signal peptide and the first of the mature protein separated by a "/". Taking *GmLac11(Glyma.04G119600)* as an example "1;Y;n4-17c23/24o569-591i": "1" means 1 transmembrane segments are predicted; "Y" means signal peptide was predicted; "n4-17c23/24o569-591i" indicate the protein is predicted to contain a signal peptide with a h-region between position 4 and 17 that is cleaved between position 23 and 24. It is followed by a non cytoplasmic loop and a TM segment between position 569 and 591, which is followed by a cytoplasmic loop.

Table S2a. Gene numbers in each phylogenetic class in soybean and *Arabidopsis thaliana*.

	Class I	Class II	Class III	Class IV	Class V	Class VI
Arabidopsis	2	4	4	3	3	1
Soybean	15	13	8	8	49	0

Table S2b. Specific gene list of each phylogenetic class of *Arabidopsis thaliana*.

Class I	Class II	Class III	Class IV	Class V	Class VI
AtLac2	*AtLac4*	*AtLac3*	*AtLac6*	*AtLac7*	*AtLac1*
AtLac17	*AtLac10*	*AtLac5*	*AtLac14*	*AtLac8*	
	AtLac11	*AtLac12*	*AtLac15*	*AtLac9*	
	AtLac16	*AtLac13*			

Table S2c. Specific gene list of each phylogenetic calss of soybean.

Class I	Class II	Class III	Class IV	Class V	Class VI
GmLac20	*GmLac2*	*GmLac7*	*GmLac18*	*GmLac1*	
GmLac21	*GmLac3*	*GmLac8*	*GmLac19*	*GmLac5*	
GmLac30	*GmLac4*	*GmLac45*	*GmLac37*	*GmLac10*	
GmLac31	*GmLac6*	*GmLac46*	*GmLac38*	*GmLac11*	
GmLac32	*GmLac9*	*GmLac58*	*GmLac47*	*GmLac12*	
GmLac33	*GmLac15*	*GmLac61*	*GmLac64*	*GmLac13*	
GmLac34	*GmLac41*	*GmLac70*	*GmLac87*	*GmLac14*	
GmLac44	*GmLac42*	*GmLac71*	*GmLac88*	*GmLac16*	
GmLac50	*GmLac52*			*GmLac17*	
GmLac56	*GmLac59*			*GmLac22*	
GmLac73	*GmLac60*			*GmLac23*	
GmLac74	*GmLac72*			*GmLac24*	
GmLac75	*GmLac81*			*GmLac25*	
GmLac76				*GmLac26*	
GmLac77				*GmLac27*	
				GmLac27	
				GmLac28	
				GmLac29	
				GmLac35	
				GmLac36	
				GmLac39	
				GmLac40	
				GmLac43	
				GmLac48	
				GmLac49	
				GmLac51	
				GmLac53	

GmLac54
GmLac55
GmLac57
GmLac62
GmLac63
GmLac65
GmLac65
GmLac66
GmLac67
GmLac68
GmLac68
GmLac69
GmLac78
GmLac79
GmLac80
GmLac82
GmLac83
GmLac84
GmLac85
GmLac86
GmLac89
GmLac90
GmLac91
GmLac92
GmLac93

Table S3. Collinear gene pairs and their Ka, Ks value and estimated divergence time.
(Abbreviations marked with an asterisk are explained at the bottom of the table)

Colinearity pair	Ka*	Ks*	Ka/Ks	PS*	MYA*
GmLac9-GmLac59	0.196368	3.91685	0.0501342	Yes	321.0532787
GmLac51-GmLac65	0.195282	3.88554	0.0502586	Yes	318.4868852
GmLac20-GmLac44	0.181731	3.32046	0.0547307	Yes	272.1688525
GmLac2-GmLac59	0.201564	3.6351	0.0554493	Yes	297.9590164
GmLac50-GmLac76	0.20168	3.32436	0.0606671	Yes	272.4885246
GmLac20-GmLac50	0.185298	2.95156	0.0627797	Yes	241.9311475
GmLac7-GmLac61	0.0124896	0.198662	0.0628684	Yes	16.28377049
GmLac44-GmLac76	0.197848	2.83032	0.069903	Yes	231.9934426
GmLac8-GmLac58	0.00958243	0.134479	0.0712559	Yes	11.02286885
GmLac58-GmLac70	0.0657891	0.921267	0.0714116	Yes	75.51368852
GmLac16-GmLac63	0.261972	3.51285	0.0745753	Yes	287.9385246
GmLac46-GmLac58	0.073337	0.982303	0.0746583	Yes	80.51663934
GmLac6-GmLac60	0.0102928	0.137023	0.0751171	Yes	11.23139344
GmLac58-GmLac61	0.272414	3.29963	0.0825591	Yes	270.4614754
GmLac8-GmLac70	0.0664403	0.800032	0.083047	Yes	65.57639344
GmLac59-GmLac81	0.241065	2.87585	0.0838237	Yes	235.7254098
GmLac7-GmLac58	0.273713	3.24728	0.0842901	Yes	266.1704918
GmLac8-GmLac46	0.0709991	0.83205	0.0853303	Yes	68.20081967
GmLac61-GmLac70	0.279239	3.22149	0.0866801	Yes	264.0565574
GmLac7-GmLac70	0.278889	3.20646	0.0869771	Yes	262.8245902
GmLac7-GmLac46	0.278412	3.19783	0.0870627	Yes	262.1172131
GmLac58-GmLac71	0.279712	3.19441	0.0875631	Yes	261.8368852
Glyma.07G146000-GmLac59	0.2787	3.17316	0.0878303	Yes	260.095082
GmLac46-GmLac61	0.280777	3.19201	0.0879624	Yes	261.6401639
GmLac45-GmLac70	0.288072	3.25489	0.0885045	Yes	266.7942623
GmLac7-GmLac8	0.287432	3.2	0.0898226	Yes	262.295082
GmLac2-GmLac9	0.0164114	0.17666	0.0928982	Yes	14.48032787
GmLac70-GmLac71	0.295454	3.17605	0.0930256	Yes	260.3319672
GmLac27-GmLac65	0.0125557	0.134181	0.0935728	Yes	10.99844262
GmLac16-GmLac51	0.0115055	0.122695	0.0937734	Yes	10.05696721
GmLac10-GmLac14	0.0113937	0.116376	0.0979039	Yes	9.539016393
GmLac9-GmLac30	0.308711	3.13059	0.0986113	Yes	256.6057377
GmLac5-GmLac40	0.0118436	0.118744	0.0997404	Yes	9.733114754
GmLac27-GmLac51	0.194104	1.91728	0.101239	Yes	157.1540984
GmLac56-GmLac76	0.348499	3.34306	0.104246	Yes	274.0213115
GmLac43-GmLac49	0.0220598	0.210407	0.104843	Yes	17.24647541
GmLac60-GmLac72	0.0609223	0.575144	0.105925	Yes	47.14295082
GmLac14-GmLac69	0.0651769	0.601309	0.108392	Yes	49.28762295
GmLac10-GmLac69	0.06673	0.601017	0.111029	Yes	49.26368852
GmLac6-GmLac72	0.0624787	0.547728	0.114069	Yes	44.8957377
GmLac3-GmLac41	0.0714705	0.623709	0.11459	Yes	51.12368852

GmLac25-GmLac82	0.0507336	0.442396	0.114679	Yes	36.26196721
GmLac45-GmLac71	0.0129563	0.111965	0.115718	Yes	9.177459016
GmLac14-GmLac62	0.0783131	0.674162	0.116164	Yes	55.25918033
GmLac20-GmLac56	0.365112	3.10799	0.117475	Yes	254.7532787
GmLac10-GmLac62	0.0792715	0.662447	0.119665	Yes	54.29893443
GmLac51-GmLac63	0.270369	2.21823	0.121885	Yes	181.8221311
GmLac11-GmLac17	0.018371	0.130036	0.141276	Yes	10.65868852
GmLac46-GmLac70	0.0282222	0.197486	0.142907	Yes	16.18737705
GmLac12-GmLac66	0.0138972	0.0959923	0.144775	Yes	7.868221311
GmLac51-GmLac53	0.0865494	0.585275	0.147878	Yes	47.97336066
GmLac20-GmLac76	0.0197777	0.132608	0.149144	Yes	10.8695082
GmLac39-GmLac86	0.0349413	0.233241	0.149808	Yes	19.11811475
GmLac62-GmLac69	0.0182972	0.119069	0.153669	Yes	9.759754098
GmLac7-GmLac45	0.0966942	0.589913	0.163913	Yes	48.35352459
GmLac16-GmLac53	0.0884867	0.53929	0.16408	Yes	44.20409836
GmLac7-GmLac71	0.0951587	0.570828	0.166703	Yes	46.78918033
GmLac1-GmLac22	0.249852	1.45384	0.171856	Yes	119.1672131
GmLac40-GmLac67	0.103556	0.596757	0.173532	Yes	48.9145082
GmLac5-GmLac67	0.0993715	0.562202	0.176754	Yes	46.08213115
GmLac1-GmLac78	0.239779	1.34105	0.178799	Yes	109.9221311
GmLac13-GmLac67	0.0315271	0.170587	0.184815	Yes	13.98254098
GmLac50-GmLac56	0.281919	1.51079	0.186603	Yes	123.8352459
GmLac31-GmLac73	0.0884371	0.473084	0.186937	Yes	38.77737705
GmLac61-GmLac71	0.0954206	0.505746	0.188673	Yes	41.45459016
GmLac55-GmLac83	0.0597235	0.314047	0.190174	Yes	25.74155738
GmLac45-GmLac61	0.0971357	0.502851	0.19317	Yes	41.21729508
GmLac9-GmLac81	0.117662	0.577507	0.203742	Yes	47.33663934
GmLac2-GmLac81	0.124407	0.601167	0.206943	Yes	49.27598361
GmLac13-GmLac40	0.109704	0.527781	0.207859	Yes	43.2607377
GmLac44-GmLac56	0.313057	1.31851	0.237432	Yes	108.0745902
GmLac44-GmLac50	0.0256811	0.106637	0.240828	Yes	8.740737705
GmLac9-Glyma.07G146000	0.213896	0.837492	0.255401	Yes	68.64688525
GmLac2-Glyma.07G146000	0.205005	0.798374	0.256778	Yes	65.4404918
GmLac15-GmLac52	0.0385949	0.130218	0.296387	Yes	10.67360656
GmLac22-GmLac78	0.0720579	0.241016	0.298975	Yes	19.75540984
GmLac27-GmLac63	0.175429	0.562948	0.311626	Yes	46.14327869
GmLac36-GmLac89	0.0768827	0.243038	0.31634	Yes	19.92114754
GmLac63-GmLac65	0.175471	0.517521	0.33906	Yes	42.4197541
Glyma.02G076300-GmLac87	0.335276	0.900699	0.37224	Yes	73.82778689
GmLac64-GmLac87	0.246166	0.649212	0.379176	Yes	53.21409836
Glyma.02G076300-GmLac37	0.348879	0.911393	0.382797	Yes	74.70434426
Glyma.07G146000-GmLac81	0.126821	0.31457	0.403155	Yes	25.78442623
GmLac37-GmLac87	0.10082	0.249998	0.403283	Yes	20.49163934
Glyma.02G076300-GmLac64	0.177794	0.308632	0.576071	Yes	25.29770492

Note: **Ks** (non-synonymous nucleotide substitution rate); **Ka** (synonymous nucleotide substitution rate); **PS** (Purifying selection); **MYA** (million years ago).

Table S4. List of Tandem repeat and Segmental repeat laccase genes. (Abbreviations marked with an asterisk are explained at the bottom of the table)

Segmental pairs	SRM*	TRM*
GmLac1—GmLac22	*GmLac1*	*GmLac3*
GmLac1—GmLac78	*GmLac2*	*GmLac4*
GmLac2—GmLac9	*GmLac3*	*GmLac18*
GmLac2—Glyma.07G146000	*GmLac5*	*GmLac19*
GmLac2—GmLac59	*GmLac6*	*GmLac20*
GmLac2—GmLac81	*GmLac7*	*GmLac21*
GmLac3—GmLac41	*GmLac8*	*GmLac22*
GmLac5—GmLac40	*GmLac9*	*GmLac23*
GmLac5—GmLac67	*GmLac10*	*GmLac24*
Glyma.02G076300—GmLac37	*GmLac11*	*GmLac25*
Glyma.02G076300—GmLac64	*GmLac12*	*GmLac26*
Glyma.02G076300—GmLac87	*GmLac13*	*GmLac31*
GmLac6—GmLac60	*GmLac14*	*GmLac32*
GmLac6—GmLac72	*GmLac15*	*GmLac33*
GmLac7—GmLac8	*GmLac16*	*GmLac34*
GmLac7—GmLac45	*GmLac20*	*GmLac37*
GmLac7—GmLac46	*GmLac22*	*GmLac38*
GmLac7—GmLac58	*GmLac25*	*GmLac41*
GmLac7—GmLac61	*GmLac27*	*GmLac42*
GmLac7—GmLac70	*GmLac31*	*GmLac67*
GmLac7—GmLac71	*GmLac36*	*GmLac68*
GmLac8—GmLac46	*GmLac37*	*GmLac73*
GmLac8—GmLac58	*GmLac39*	*GmLac74*
GmLac8—GmLac70	*GmLac40*	*GmLac75*
GmLac9—Glyma.07G146000	*GmLac43*	*GmLac76*
GmLac9—GmLac30	*GmLac44*	*GmLac77*
GmLac9—GmLac59	*GmLac45*	*GmLac78*
GmLac9—GmLac81	*GmLac46*	*GmLac79*
GmLac10—GmLac14	*GmLac50*	*GmLac80*
GmLac10—GmLac62	*GmLac51*	*GmLac83*
GmLac10—GmLac69	*GmLac55*	*GmLac84*
GmLac11—GmLac17	*GmLac56*	*GmLac85*
GmLac12—GmLac66	*GmLac58*	*GmLac87*
GmLac13—GmLac40	*GmLac59*	*GmLac88*
GmLac13—GmLac67	*GmLac60*	*GmLac89*
GmLac14—GmLac62	*GmLac61*	*GmLac90*
GmLac14—GmLac69	*GmLac62*	*GmLac91*
GmLac15—GmLac52	*GmLac63*	*GmLac92*
GmLac16—GmLac51	*GmLac64*	
GmLac16—GmLac53	*GmLac70*	

GmLac16—GmLac63	*Glyma.02G076300*
GmLac20—GmLac44	*Glyma.07G146000*
GmLac20—GmLac50	
GmLac20—GmLac56	
GmLac20—GmLac76	
GmLac22—GmLac78	
Glyma.07G146000—GmLac59	
Glyma.07G146000—GmLac81	
GmLac25—GmLac82	
GmLac27—GmLac51	
GmLac27—GmLac63	
GmLac27—GmLac65	
GmLac31—GmLac73	
GmLac36—GmLac89	
GmLac37—GmLac87	
GmLac39—GmLac86	
GmLac40—GmLac67	
GmLac43—GmLac49	
GmLac44—GmLac50	
GmLac44—GmLac56	
GmLac44—GmLac76	
GmLac45—GmLac61	
GmLac45—GmLac70	
GmLac45—GmLac71	
GmLac46—GmLac58	
GmLac46—GmLac61	
GmLac46—GmLac70	
GmLac50—GmLac56	
GmLac50—GmLac76	
GmLac51—GmLac53	
GmLac51—GmLac63	
GmLac51—GmLac65	
GmLac55—GmLac83	
GmLac56—GmLac76	
GmLac58—GmLac61	
GmLac58—GmLac70	
GmLac58—GmLac71	
GmLac59—GmLac81	
GmLac60—GmLac72	
GmLac61—GmLac70	
GmLac61—GmLac71	
GmLac62—GmLac69	
GmLac63—GmLac65	
GmLac64—GmLac87	

GmLac70—GmLac71

Note: **SRM** (Segmental repeat members) indicated laccase gene family member’s invoved in segmental dupulation; **TRM** (Tandem repeat members) indicate laccase gene family members that show tandem repeat on a chromosome.

Figure S1. Schematic diagram of the motifs of soybean laccase proteins.

The schematic diagram was derived from MEME. The order of motifs of laccase proteins in the diagram was automatically generated by MEME according to their score values.

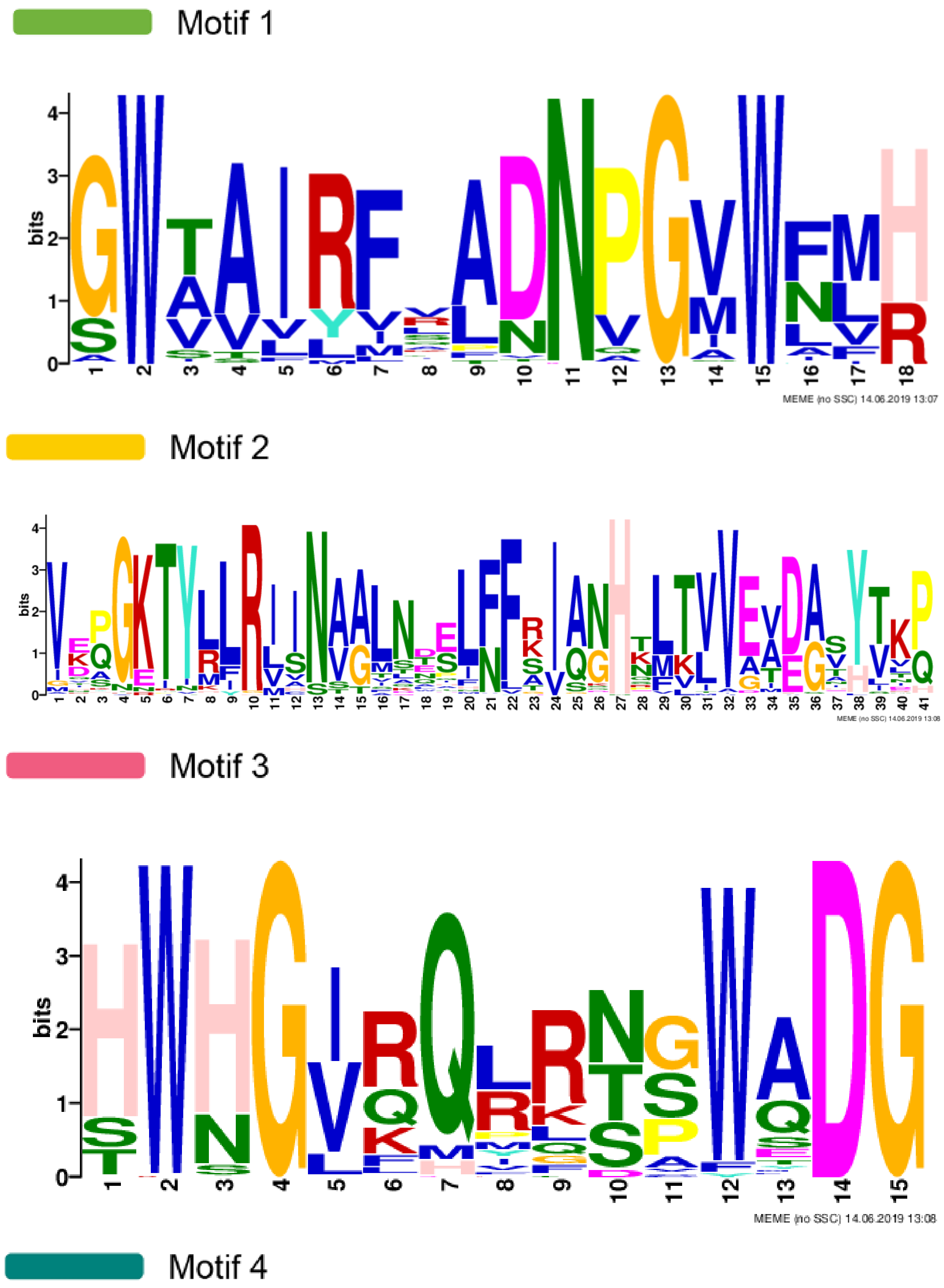

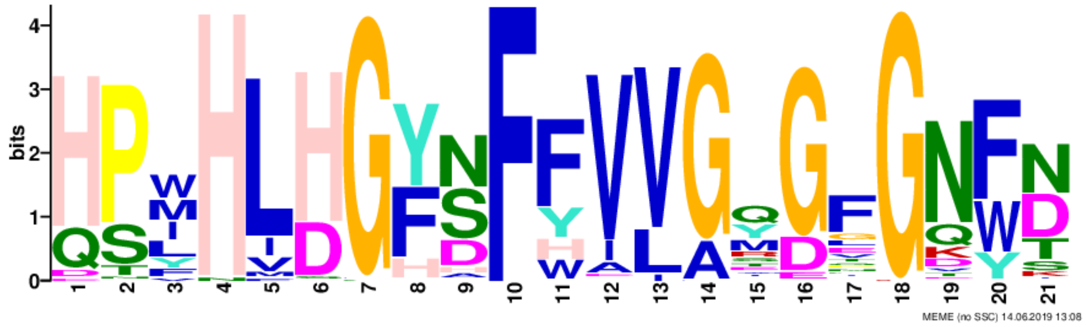

Motif 5

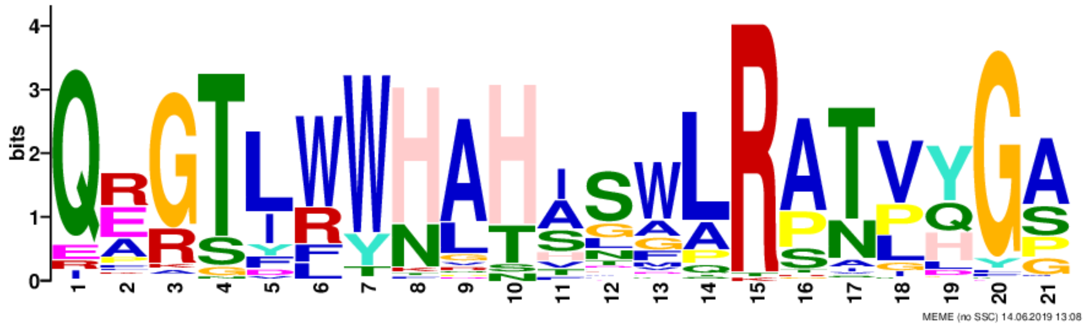

Motif 6

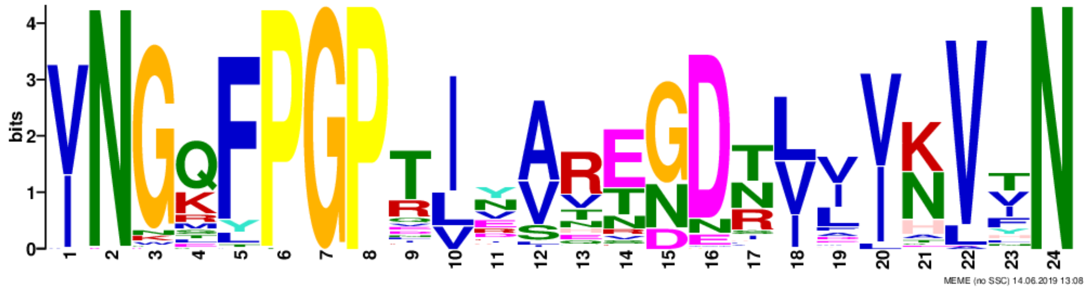

Motif 7

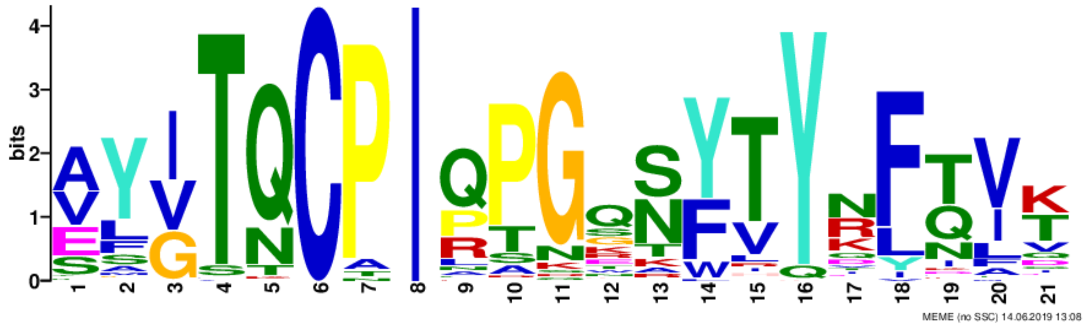

Motif 8

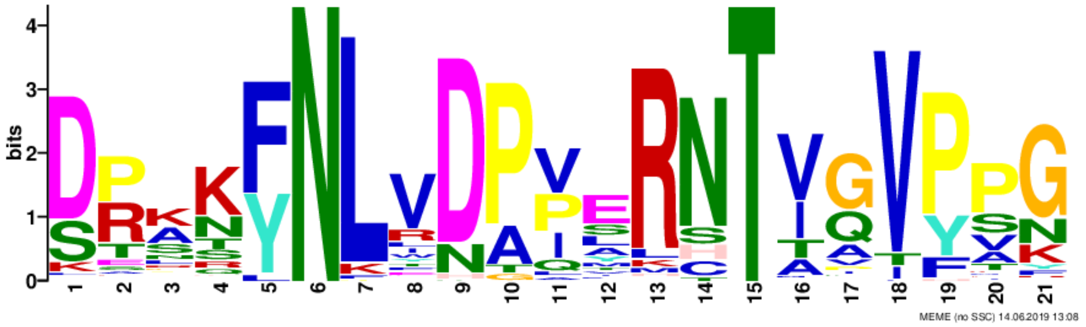

Motif 9

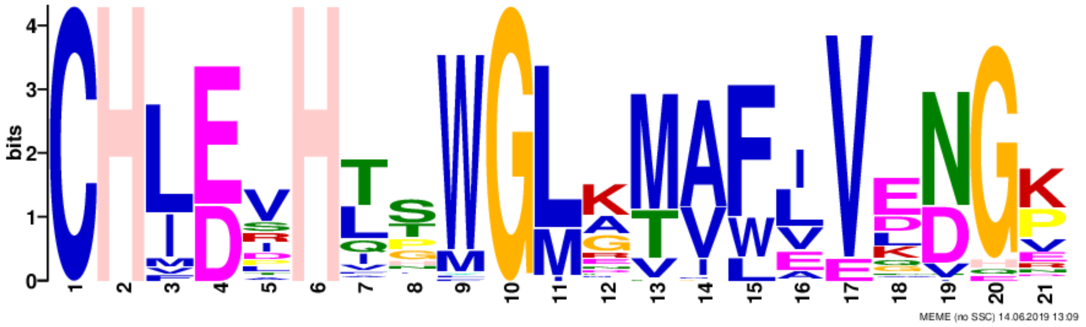

Motif 10

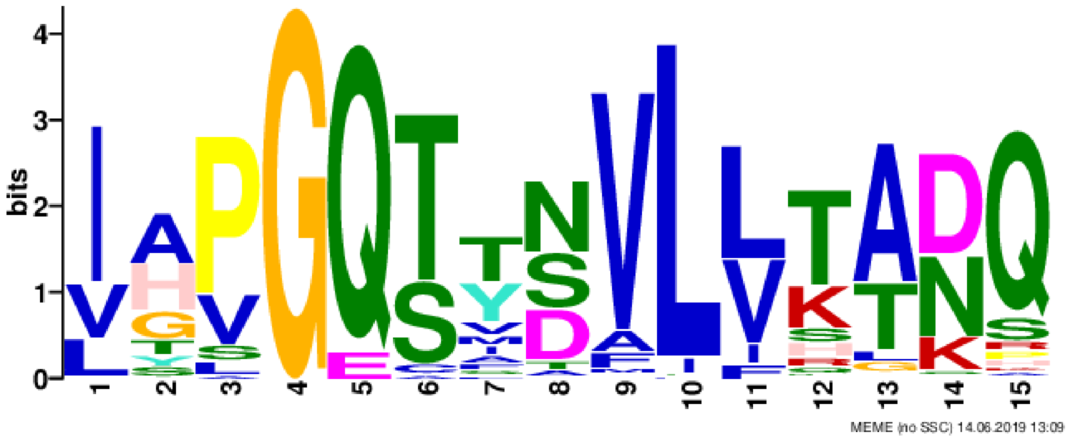

Table S5a. Expression pattern of soybean laccase gene family. (Abbreviations marked with an asterisk and expression values are explained at the bottom of the table)

Gene name	Root	RH*	Nodules	Pod	Seed	Stem	SAM*	Leaves	Flower	MV1*	Scale (value)*
GmLac1	41.64	4.94	6.82	0.06	0.00	0.04	0.01	0.03	0.14	5.96	0
GmLac2	0.94	6.30	12.58	10.45	0.45	70.53	3.31	5.93	1.92	12.49	5
GmLac3	0.64	0.78	1.38	0.14	0.17	0.17	0.24	0.20	0.21	0.44	10
GmLac4	1.33	1.20	3.37	1.41	2.90	2.27	2.20	1.62	1.68	2.00	15
GmLac5	29.87	66.04	33.99	37.23	14.55	46.41	24.14	2.00	6.78	29.00	20
GmLac6	1.67	3.92	8.33	0.68	0.04	17.82	1.37	1.64	0.26	3.97	25
GmLac7	3.43	13.10	18.70	0.08	0.15	0.39	0.16	0.02	0.34	4.04	30
GmLac8	2.95	4.36	3.94	7.97	0.69	32.58	0.56	0.98	1.91	6.22	
GmLac9	1.00	7.28	15.93	10.78	0.61	75.27	3.25	5.62	2.18	13.55	
GmLac10	11.59	44.89	11.61	73.40	111.75	53.38	23.71	2.26	11.45	38.23	
GmLac11	8.69	4.61	5.28	7.81	14.03	6.46	5.03	2.10	0.79	6.09	
GmLac12	54.73	65.77	60.52	23.36	22.16	47.63	55.08	6.07	5.52	37.87	
GmLac13	0.02	0.10	0.27	0.19	0.12	2.07	0.06	0.05	0.05	0.33	
GmLac14	9.63	30.22	10.13	10.23	16.41	32.70	10.80	0.95	3.62	13.85	
GmLac15	0.00	0.04	0.13	0.16	0.00	0.17	0.02	0.04	0.07	0.07	
GmLac16	36.50	49.47	10.29	129.80	4.81	99.05	114.40	14.28	18.43	53.00	
GmLac17	3.59	2.72	3.28	3.18	5.06	3.80	2.48	0.75	1.27	2.90	
GmLac18	0.09	0.69	0.14	0.11	2.53	0.09	3.88	0.00	0.09	0.85	
GmLac19	0.11	0.51	0.10	0.54	0.51	1.01	0.73	0.55	0.04	0.46	
GmLac20	0.06	4.26	5.34	18.08	0.01	66.65	0.08	0.64	0.12	10.58	
GmLac21	0.57	3.16	4.30	54.10	0.12	36.46	0.64	2.81	0.56	11.41	
GmLac22	0.43	0.97	3.16	0.30	0.04	5.16	0.52	1.21	0.17	1.33	
GmLac23	0.02	0.00	0.00	5.35	6.87	0.00	0.04	0.01	0.52	1.42	

GmLac24	38.53	11.66	21.13	0.08	0.00	0.03	0.09	2.86	0.21	8.29
GmLac25	0.20	0.00	0.00	0.01	0.00	1.06	0.20	0.03	505.03	56.28
GmLac26	0.00	0.00	0.00	0.00	0.00	0.06	0.00	0.00	22.85	2.54
GmLac27	13.26	23.59	7.66	8.59	6.77	3.95	17.17	0.41	1.85	9.25
GmLac28	0.00	0.16	1.40	0.00	0.00	0.00	0.00	0.03	0.00	0.18
GmLac29	4.53	8.33	6.05	11.75	10.70	13.51	13.12	3.33	3.38	8.30
GmLac30	0.00	0.01	0.00	0.01	0.00	0.02	0.10	0.00	0.02	0.02
GmLac31	0.91	3.45	6.42	3.37	0.01	15.23	4.51	1.76	1.10	4.08
GmLac32	0.00	0.06	0.03	1.92	0.03	0.40	0.00	0.05	0.03	0.28
GmLac33	0.53	0.70	0.66	0.78	0.56	3.97	0.51	0.39	0.26	0.93
GmLac34	0.00	0.04	0.01	0.02	0.04	0.03	0.04	0.00	0.00	0.02
GmLac35	0.03	0.00	0.00	0.00	0.00	0.01	0.00	0.00	0.01	0.01
GmLac36	0.00	0.00	0.04	0.02	0.00	0.02	0.00	0.00	0.09	0.02
GmLac37	3.78	0.02	0.04	0.11	7.01	0.79	23.46	0.12	0.79	4.01
GmLac38	0.20	0.00	0.00	0.09	0.18	0.13	4.43	0.00	0.35	0.60
GmLac39	0.76	2.29	0.92	8.24	4.87	3.74	3.60	11.39	3.14	4.33
GmLac40	33.53	59.28	40.51	33.92	10.26	87.08	43.94	6.10	9.01	35.96
GmLac41	0.63	0.98	3.94	0.93	0.58	1.83	1.53	1.66	0.90	1.44
GmLac42	1.56	2.07	6.79	1.25	0.04	0.76	0.26	0.19	0.31	1.47
GmLac43	3.11	2.62	1.38	2.35	3.16	5.41	0.85	2.11	3.56	2.73
GmLac44	0.05	0.18	0.39	219.15	0.01	0.31	0.07	0.16	2.36	24.74
GmLac45	0.70	0.05	0.03	6.21	0.04	1.64	16.02	0.00	0.82	2.84
GmLac46	3.38	3.71	3.93	71.19	0.07	42.16	0.87	5.17	1.15	14.63
GmLac47	0.02	0.01	0.05	0.74	2.75	0.00	0.01	0.00	0.01	0.40
GmLac48	0.00	0.02	0.03	0.01	0.01	0.01	0.00	0.00	0.73	0.09
GmLac49	5.96	1.85	2.96	0.42	0.22	1.84	0.46	0.66	1.47	1.76

GmLac50	0.25	2.16	3.29	45.74	0.20	6.87	0.42	1.29	0.74	6.77
GmLac51	14.63	19.92	7.13	60.15	3.77	61.18	61.51	7.11	12.99	27.60
GmLac52	0.18	0.59	0.63	4.39	0.46	1.15	0.01	0.23	0.10	0.86
GmLac53	3.08	7.99	5.15	2.43	2.66	2.09	1.86	0.21	0.63	2.90
GmLac54	0.01	0.00	0.00	0.00	0.03	0.02	0.00	0.00	0.10	0.02
GmLac55	162.50	50.75	30.23	12.38	3.09	36.75	8.77	12.19	4.63	35.70
GmLac56	0.00	0.00	0.00	1.37	0.00	0.00	0.21	0.00	0.02	0.18
GmLac57	0.19	0.00	0.00	0.21	0.90	0.02	17.93	0.28	2.90	2.49
GmLac58	0.74	7.17	7.19	7.00	0.08	66.23	1.79	0.23	0.70	10.12
GmLac59	0.44	3.74	9.64	15.62	0.01	40.78	1.54	5.87	1.01	8.74
GmLac60	1.90	3.53	8.79	0.98	0.03	5.67	0.55	1.62	0.30	2.60
GmLac61	1.33	8.78	14.21	0.55	0.11	0.90	0.08	0.16	1.45	3.06
GmLac62	27.32	53.26	32.22	21.12	15.68	33.04	8.42	1.00	2.10	21.57
GmLac63	0.00	0.00	0.00	0.00	0.00	0.02	0.02	0.00	0.04	0.01
GmLac64	1.08	0.38	1.11	29.43	220.49	0.42	0.68	0.27	0.65	28.28
GmLac65	69.51	127.58	54.73	26.11	33.73	26.31	62.91	1.39	4.16	45.16
GmLac66	66.83	56.05	48.36	56.52	35.88	84.13	43.27	8.40	9.79	45.47
GmLac67	6.64	15.71	22.65	33.72	1.07	206.44	32.88	0.43	5.14	36.08
GmLac68	64.91	47.40	37.21	0.05	0.02	0.03	0.01	0.00	0.03	16.63
GmLac69	47.09	65.88	29.72	2.73	0.36	28.53	16.04	3.85	4.14	22.04
GmLac70	2.02	5.15	3.85	131.31	0.02	31.12	0.37	2.15	1.16	19.68
GmLac71	0.26	0.02	0.00	1.05	0.00	0.90	11.92	0.00	0.21	1.59
GmLac72	0.59	1.08	1.32	0.15	0.06	10.85	1.10	1.00	0.29	1.83
GmLac73	0.16	0.37	0.78	1.51	0.07	3.05	0.41	0.07	0.08	0.72
GmLac74	0.17	0.68	1.18	2.78	0.14	4.58	0.81	0.21	0.18	1.19
GmLac75	0.07	0.19	0.09	0.32	0.00	0.26	1.14	0.36	0.17	0.29

GmLac76	0.00	1.32	1.54	2.35	0.00	20.37	0.01	0.14	0.03	2.86
GmLac77	0.16	1.65	2.13	35.66	0.05	16.90	0.30	1.16	0.32	6.48
GmLac78	0.10	0.02	0.08	0.00	0.02	0.00	0.00	0.00	0.00	0.02
GmLac79	0.13	0.01	0.00	0.07	0.09	0.00	0.04	0.04	0.14	0.06
GmLac80	347.87	16.01	30.92	0.31	0.00	0.11	0.00	9.05	0.70	45.00
GmLac81	0.06	0.48	1.09	8.98	0.00	10.15	0.05	0.05	0.22	2.34
GmLac82	0.00	0.00	0.00	0.00	0.00	0.02	0.00	0.00	19.59	2.18
GmLac83	0.03	0.19	0.47	0.06	0.09	0.08	0.00	0.02	0.27	0.14
GmLac84	23.66	2.72	2.76	0.15	0.09	1.48	0.06	0.14	0.33	3.49
GmLac85	93.65	67.15	30.28	32.47	44.21	48.51	13.49	12.04	2.55	38.26
GmLac86	1.25	5.09	6.20	10.36	17.20	28.81	6.63	23.16	10.68	12.15
GmLac87	13.60	0.03	0.00	0.00	0.01	2.87	117.91	0.00	2.54	15.22
GmLac88	1.57	0.00	0.00	0.00	0.00	0.29	12.32	0.00	0.09	1.59
GmLac89	4.44	0.28	0.10	0.04	0.05	0.09	0.04	0.00	2.18	0.80
GmLac90	0.02	0.06	0.27	0.02	0.00	0.00	0.12	0.00	0.11	0.07
MV2*	14.22	11.22	7.99	14.61	7.02	17.39	9.00	2.00	7.90	10.15

Note: **RH** (Root hairs); **SAM** (Stem apical meristem); **MV1** (Mean value1) indicate the average expression value of a gene in nine tissues/organs; **MV2** (Mean value 2) indicate the average expression value of 90 laccase genes in a one tissue/organ; **Scale (value)** a scale used to indicate different expression abundance, use light to dark red represent different expression abundance; Expression values are based **FPKM** (Fragments per Kilobase Million). We difine 3-fold of average value (10.15) of 90 genes in 9 tissues/organs as high expression gene.

Table S5b. Genes that are barely expressed in 9 detectet materials.

Gene name	Root	RH	Nodules	Pod	Seed	Stem	SAM	Leaves	Flower
GmLac34	0.00	0.04	0.01	0.02	0.04	0.03	0.04	0.00	0.00
GmLac35	0.03	0.00	0.00	0.00	0.00	0.01	0.00	0.00	0.01
GmLac36	0.00	0.00	0.04	0.02	0.00	0.02	0.00	0.00	0.09
GmLac48	0.00	0.02	0.03	0.01	0.01	0.01	0.00	0.00	0.73
GmLac54	0.01	0.00	0.00	0.00	0.03	0.02	0.00	0.00	0.10
GmLac63	0.00	0.00	0.00	0.00	0.00	0.02	0.02	0.00	0.04
GmLac78	0.10	0.02	0.08	0.00	0.02	0.00	0.00	0.00	0.00
GmLac79	0.13	0.01	0.00	0.07	0.09	0.00	0.04	0.04	0.14
GmLac83	0.03	0.19	0.47	0.06	0.09	0.08	0.00	0.02	0.27
GmLac90	0.02	0.06	0.27	0.02	0.00	0.00	0.12	0.00	0.11

Note: Refer to Additional file 6 (sheet 1).

Table S6. Comparison of expression patterns between colinearity pairs. (Abbreviation explanation refer to Additional file 6, other declares listed at the bottom of the table)

Colinearity pairs	Gene name	Root	RH*	Nodules	Pod	Seed	Stem	SAM*	Leaves	Flower
Glyma.02G076300-	*Glyma.02G076300*	#N/A	#N/A	#N/A	#N/A	#N/A	#N/A	#N/A	#N/A	#N/A
GmLac37	*GmLac37*	3.77682	0.02212	0.03889	0.105609	7.01387	0.79394	23.4562	0.11764	0.7901
Glyma.02G076300-	*Glyma.02G076300*	#N/A	#N/A	#N/A	#N/A	#N/A	#N/A	#N/A	#N/A	#N/A
GmLac64	*GmLac64*	1.07807	0.38154	1.11208	29.4318	220.487	0.42315	0.67943	0.27023	0.65259
Glyma.02G076300-	*Glyma.02G076300*	#N/A	#N/A	#N/A	#N/A	#N/A	#N/A	#N/A	#N/A	#N/A
GmLac87	*GmLac87*	13.599	0.02908	0	0	0.009975	2.87219	117.911	0	2.53511
Glyma.07G146000-	*Glyma.07G146000*	#N/A	#N/A	#N/A	#N/A	#N/A	#N/A	#N/A	#N/A	#N/A
GmLac59	*GmLac59*	0.43981	3.74448	9.644	15.6223	0.009872	40.7784	1.54436	5.86649	1.01082
Glyma.07G146000-	*Glyma.07G146000*	#N/A	#N/A	#N/A	#N/A	#N/A	#N/A	#N/A	#N/A	#N/A
GmLac81	*GmLac81*	0.05663	0.48416	1.08664	8.97794	0	10.1503	0.04955	0.05114	0.22449
GmLac10-GmLac14	*GmLac10*	11.5899	44.8886	11.6101	73.4047	111.748	53.3777	23.7094	2.26299	11.447
	GmLac14	9.63394	30.2187	10.13	10.2349	16.4061	32.6984	10.7951	0.94599	3.6231
GmLac10-GmLac62	*GmLac10*	11.5899	44.8886	11.6101	73.4047	111.748	53.3777	23.7094	2.26299	11.447
	GmLac62	27.3235	53.2608	32.2232	21.1231	15.6818	33.0403	8.41675	0.99737	2.09606
GmLac10-GmLac69	*GmLac10*	11.5899	44.8886	11.6101	73.4047	111.748	53.3777	23.7094	2.26299	11.447
	GmLac69	47.0863	65.8791	29.7196	2.73122	0.358669	28.528	16.0405	3.84799	4.1437
GmLac11-GmLac17	*GmLac11*	8.68507	4.6075	5.27789	7.81244	14.0267	6.45699	5.03375	2.10009	0.78851
	GmLac17	3.58944	2.71941	3.28167	3.1781	5.06165	3.79891	2.47571	0.75273	1.2702
GmLac12-GmLac66	*GmLac12*	54.7267	65.7731	60.5153	23.3617	22.164	47.6317	55.0847	6.0687	5.52321
	GmLac66	66.8345	56.0486	48.3648	56.5199	35.877	84.13	43.2721	8.3978	9.78837
GmLac13-GmLac40	*GmLac13*	0.02002	0.10168	0.27329	0.189668	0.123885	2.06787	0.06217	0.05427	0.05264
	GmLac40	33.5331	59.2758	40.5081	33.9158	10.2627	87.0806	43.9428	6.09931	9.00949
GmLac13-GmLac67	*GmLac13*	0.02002	0.10168	0.27329	0.189668	0.123885	2.06787	0.06217	0.05427	0.05264

GmLac14-GmLac62	*GmLac67*	6.64409	15.7084	22.6459	33.7204	1.0697	206.436	32.8812	0.42912	5.14144
	GmLac14	9.63394	30.2187	10.13	10.2349	16.4061	32.6984	10.7951	0.94599	3.6231
	GmLac62	27.3235	53.2608	32.2232	21.1231	15.6818	33.0403	8.41675	0.99737	2.09606
GmLac14-GmLac69	*GmLac14*	9.63394	30.2187	10.13	10.2349	16.4061	32.6984	10.7951	0.94599	3.6231
	GmLac69	47.0863	65.8791	29.7196	2.73122	0.358669	28.528	16.0405	3.84799	4.1437
GmLac15-GmLac52	*GmLac15*	0	0.03701	0.12832	0.163823	0	0.16703	0.02097	0.04407	0.06766
	GmLac52	0.18233	0.58785	0.62554	4.38931	0.458937	1.15019	0.01074	0.23023	0.10126
GmLac16-GmLac51	*GmLac16*	36.498	49.4705	10.2934	129.799	4.80801	99.0501	114.403	14.2751	18.4324
	GmLac51	14.6344	19.9245	7.12984	60.1506	3.77382	61.1832	61.508	7.11307	12.9894
GmLac16-GmLac53	*GmLac16*	36.498	49.4705	10.2934	129.799	4.80801	99.0501	114.403	14.2751	18.4324
	GmLac53	3.07529	7.9933	5.14657	2.42602	2.65726	2.08877	1.85632	0.21466	0.62825
GmLac16-GmLac63	*GmLac16*	36.498	49.4705	10.2934	129.799	4.80801	99.0501	114.403	14.2751	18.4324
	GmLac63	0	0	0	0	0	0.0168	0.01694	0	0.04252
GmLac1-GmLac22	*GmLac1*	41.6351	4.94473	6.82357	0.061812	0	0.03739	0.01244	0.02541	0.13736
	GmLac22	0.43127	0.96933	3.16485	0.303696	0.040318	5.16388	0.51652	1.211	0.17482
GmLac1-GmLac78	*GmLac1*	41.6351	4.94473	6.82357	0.061812	0	0.03739	0.01244	0.02541	0.13736
	GmLac78	0.09805	0.02228	0.07835	0	0.022626	0	0	0	0
GmLac20-GmLac44	*GmLac20*	0.05868	4.25552	5.33585	18.0807	0.009057	66.646	0.0836	0.63572	0.12106
	GmLac44	0.04611	0.18383	0.39179	219.151	0.009614	0.30732	0.07063	0.15598	2.35643
GmLac20-GmLac50	*GmLac20*	0.05868	4.25552	5.33585	18.0807	0.009057	66.646	0.0836	0.63572	0.12106
	GmLac50	0.2465	2.16443	3.28555	45.742	0.19514	6.86754	0.41932	1.29423	0.74085
GmLac20-GmLac56	*GmLac20*	0.05868	4.25552	5.33585	18.0807	0.009057	66.646	0.0836	0.63572	0.12106
	GmLac56	0	0	0	1.37444	0	0	0.20844	0	0.02231
GmLac20-GmLac76	*GmLac20*	0.05868	4.25552	5.33585	18.0807	0.009057	66.646	0.0836	0.63572	0.12106
	GmLac76	0	1.31579	1.54197	2.35281	0	20.3688	0.01065	0.14062	0.03435
GmLac22-GmLac78	*GmLac22*	0.43127	0.96933	3.16485	0.303696	0.040318	5.16388	0.51652	1.211	0.17482

	GmLac78	0.09805	0.02228	0.07835	0	0.022626	0	0	0	0
GmLac25-GmLac82	*GmLac25*	0.19815	0	0	0.006065	0	1.05872	0.19917	0.0324	505.028
	GmLac82	0	0	0	0	0	0.02196	0	0	19.5856
GmLac27-GmLac51	*GmLac27*	13.2612	23.5916	7.66377	8.58861	6.76724	3.94651	17.1658	0.40598	1.85107
	GmLac51	14.6344	19.9245	7.12984	60.1506	3.77382	61.1832	61.508	7.11307	12.9894
GmLac27-GmLac63	*GmLac27*	13.2612	23.5916	7.66377	8.58861	6.76724	3.94651	17.1658	0.40598	1.85107
	GmLac63	0	0	0	0	0	0.0168	0.01694	0	0.04252
GmLac27-GmLac65	*GmLac27*	13.2612	23.5916	7.66377	8.58861	6.76724	3.94651	17.1658	0.40598	1.85107
	GmLac65	69.5094	127.582	54.7256	26.1075	33.7318	26.3119	62.9141	1.38782	4.15973
GmLac2-	*GmLac2*	0.93778	6.29774	12.5775	10.4539	0.446109	70.5273	3.30847	5.92531	1.92027
Glyma.07G146000	*Glyma.07G146000*	#N/A	#N/A	#N/A	#N/A	#N/A	#N/A	#N/A	#N/A	#N/A
GmLac2-GmLac59	*GmLac2*	0.93778	6.29774	12.5775	10.4539	0.446109	70.5273	3.30847	5.92531	1.92027
	GmLac59	0.43981	3.74448	9.644	15.6223	0.009872	40.7784	1.54436	5.86649	1.01082
GmLac2-GmLac81	*GmLac2*	0.93778	6.29774	12.5775	10.4539	0.446109	70.5273	3.30847	5.92531	1.92027
	GmLac81	0.05663	0.48416	1.08664	8.97794	0	10.1503	0.04955	0.05114	0.22449
GmLac2-GmLac9	*GmLac2*	0.93778	6.29774	12.5775	10.4539	0.446109	70.5273	3.30847	5.92531	1.92027
	GmLac9	1.00406	7.28371	15.9315	10.7808	0.614239	75.2652	3.25306	5.61638	2.1843
GmLac31-GmLac73	*GmLac31*	0.90632	3.45205	6.41611	3.36955	0.009214	15.2291	4.50745	1.76054	1.09615
	GmLac73	0.15953	0.3692	0.78328	1.50807	0.068867	3.05038	0.40712	0.07094	0.08397
GmLac36-GmLac89	*GmLac36*	0	0	0.03789	0.020697	0	0.02498	0	0	0.08961
	GmLac89	4.43886	0.27797	0.09775	0.039624	0.054457	0.09208	0.0395	0	2.18046
GmLac37-GmLac87	*GmLac37*	3.77682	0.02212	0.03889	0.105609	7.01387	0.79394	23.4562	0.11764	0.7901
	GmLac87	13.599	0.02908	0	0	0.009975	2.87219	117.911	0	2.53511
GmLac39-GmLac86	*GmLac39*	0.76328	2.28925	0.91852	8.24112	4.8665	3.74118	3.59805	11.3878	3.13858
	GmLac86	1.24634	5.09379	6.20044	10.3649	17.2005	28.81	6.62957	23.1612	10.6752
GmLac3-GmLac41	*GmLac3*	0.64027	0.77805	1.3811	0.137099	0.174104	0.16974	0.24305	0.19788	0.20581

	GmLac41	0.62883	0.97714	3.94434	0.933297	0.583836	1.82749	1.53321	1.65908	0.89617
GmLac40-GmLac67	*GmLac40*	33.5331	59.2758	40.5081	33.9158	10.2627	87.0806	43.9428	6.09931	9.00949
	GmLac67	6.64409	15.7084	22.6459	33.7204	1.0697	206.436	32.8812	0.42912	5.14144
GmLac43-GmLac49	*GmLac43*	3.1083	2.62313	1.37643	2.34784	3.15556	5.40909	0.84527	2.10894	3.56281
	GmLac49	5.96154	1.85364	2.95741	0.416756	0.216855	1.84117	0.46237	0.65547	1.46521
GmLac44-GmLac50	*GmLac44*	0.04611	0.18383	0.39179	219.151	0.009614	0.30732	0.07063	0.15598	2.35643
	GmLac50	0.2465	2.16443	3.28555	45.742	0.19514	6.86754	0.41932	1.29423	0.74085
GmLac44-GmLac56	*GmLac44*	0.04611	0.18383	0.39179	219.151	0.009614	0.30732	0.07063	0.15598	2.35643
	GmLac56	0	0	0	1.37444	0	0	0.20844	0	0.02231
GmLac44-GmLac76	*GmLac44*	0.04611	0.18383	0.39179	219.151	0.009614	0.30732	0.07063	0.15598	2.35643
	GmLac76	0	1.31579	1.54197	2.35281	0	20.3688	0.01065	0.14062	0.03435
GmLac45-GmLac61	*GmLac45*	0.70011	0.04702	0.03301	6.21115	0.038505	1.63832	16.0232	0	0.82492
	GmLac61	1.33023	8.77965	14.2139	0.552605	0.114535	0.90016	0.07608	0.15593	1.45204
GmLac45-GmLac70	*GmLac45*	0.70011	0.04702	0.03301	6.21115	0.038505	1.63832	16.0232	0	0.82492
	GmLac70	2.01994	5.1544	3.85343	131.307	0.015703	31.122	0.3685	2.14749	1.1624
GmLac45-GmLac71	*GmLac45*	0.70011	0.04702	0.03301	6.21115	0.038505	1.63832	16.0232	0	0.82492
	GmLac71	0.25852	0.01825	0	1.04685	0	0.90019	11.9166	0	0.20754
GmLac46-GmLac58	*GmLac46*	3.38403	3.71224	3.92638	71.1852	0.07004	42.1577	0.8744	5.17488	1.1463
	GmLac58	0.74271	7.17297	7.1864	6.99751	0.075354	66.2303	1.78565	0.23115	0.69734
GmLac46-GmLac61	*GmLac46*	3.38403	3.71224	3.92638	71.1852	0.07004	42.1577	0.8744	5.17488	1.1463
	GmLac61	1.33023	8.77965	14.2139	0.552605	0.114535	0.90016	0.07608	0.15593	1.45204
GmLac46-GmLac70	*GmLac46*	3.38403	3.71224	3.92638	71.1852	0.07004	42.1577	0.8744	5.17488	1.1463
	GmLac70	2.01994	5.1544	3.85343	131.307	0.015703	31.122	0.3685	2.14749	1.1624
GmLac50-GmLac56	*GmLac50*	0.2465	2.16443	3.28555	45.742	0.19514	6.86754	0.41932	1.29423	0.74085
	GmLac56	0	0	0	1.37444	0	0	0.20844	0	0.02231
GmLac50-GmLac76	*GmLac50*	0.2465	2.16443	3.28555	45.742	0.19514	6.86754	0.41932	1.29423	0.74085

	GmLac76	0	1.31579	1.54197	2.35281	0	20.3688	0.01065	0.14062	0.03435
GmLac51-GmLac53	*GmLac51*	14.6344	19.9245	7.12984	60.1506	3.77382	61.1832	61.508	7.11307	12.9894
	GmLac53	3.07529	7.9933	5.14657	2.42602	2.65726	2.08877	1.85632	0.21466	0.62825
GmLac51-GmLac63	*GmLac51*	14.6344	19.9245	7.12984	60.1506	3.77382	61.1832	61.508	7.11307	12.9894
	GmLac63	0	0	0	0	0	0.0168	0.01694	0	0.04252
GmLac51-GmLac65	*GmLac51*	14.6344	19.9245	7.12984	60.1506	3.77382	61.1832	61.508	7.11307	12.9894
	GmLac65	69.5094	127.582	54.7256	26.1075	33.7318	26.3119	62.9141	1.38782	4.15973
GmLac55-GmLac83	*GmLac55*	162.496	50.7464	30.2307	12.3806	3.08989	36.7526	8.76674	12.1943	4.62867
	GmLac83	0.03273	0.19458	0.47356	0.05701	0.091618	0.08484	0	0.0178	0.26555
GmLac56-GmLac76	*GmLac56*	0	0	0	1.37444	0	0	0.20844	0	0.02231
	GmLac76	0	1.31579	1.54197	2.35281	0	20.3688	0.01065	0.14062	0.03435
GmLac58-GmLac61	*GmLac58*	0.74271	7.17297	7.1864	6.99751	0.075354	66.2303	1.78565	0.23115	0.69734
	GmLac61	1.33023	8.77965	14.2139	0.552605	0.114535	0.90016	0.07608	0.15593	1.45204
GmLac58-GmLac70	*GmLac58*	0.74271	7.17297	7.1864	6.99751	0.075354	66.2303	1.78565	0.23115	0.69734
	GmLac70	2.01994	5.1544	3.85343	131.307	0.015703	31.122	0.3685	2.14749	1.1624
GmLac58-GmLac71	*GmLac58*	0.74271	7.17297	7.1864	6.99751	0.075354	66.2303	1.78565	0.23115	0.69734
	GmLac71	0.25852	0.01825	0	1.04685	0	0.90019	11.9166	0	0.20754
GmLac59-GmLac81	*GmLac59*	0.43981	3.74448	9.644	15.6223	0.009872	40.7784	1.54436	5.86649	1.01082
	GmLac81	0.05663	0.48416	1.08664	8.97794	0	10.1503	0.04955	0.05114	0.22449
GmLac5-GmLac40	*GmLac5*	29.8675	66.0376	33.9908	37.2338	14.5517	46.413	24.14	2.00479	6.7772
	GmLac40	33.5331	59.2758	40.5081	33.9158	10.2627	87.0806	43.9428	6.09931	9.00949
GmLac5-GmLac67	*GmLac5*	29.8675	66.0376	33.9908	37.2338	14.5517	46.413	24.14	2.00479	6.7772
	GmLac67	6.64409	15.7084	22.6459	33.7204	1.0697	206.436	32.8812	0.42912	5.14144
GmLac60-GmLac72	*GmLac60*	1.90439	3.5269	8.78535	0.979661	0.027152	5.6743	0.55415	1.62477	0.29559
	GmLac72	0.59074	1.08296	1.3223	0.153116	0.056937	10.8463	1.09876	1.00401	0.2863
GmLac61-GmLac70	*GmLac61*	1.33023	8.77965	14.2139	0.552605	0.114535	0.90016	0.07608	0.15593	1.45204

	GmLac70	2.01994	5.1544	3.85343	131.307	0.015703	31.122	0.3685	2.14749	1.1624
GmLac61-GmLac71	*GmLac61*	1.33023	8.77965	14.2139	0.552605	0.114535	0.90016	0.07608	0.15593	1.45204
	GmLac71	0.25852	0.01825	0	1.04685	0	0.90019	11.9166	0	0.20754
GmLac62-GmLac69	*GmLac62*	27.3235	53.2608	32.2232	21.1231	15.6818	33.0403	8.41675	0.99737	2.09606
	GmLac69	47.0863	65.8791	29.7196	2.73122	0.358669	28.528	16.0405	3.84799	4.1437
GmLac63-GmLac65	*GmLac63*	0	0	0	0	0	0.0168	0.01694	0	0.04252
	GmLac65	69.5094	127.582	54.7256	26.1075	33.7318	26.3119	62.9141	1.38782	4.15973
GmLac64-GmLac87	*GmLac64*	1.07807	0.38154	1.11208	29.4318	220.487	0.42315	0.67943	0.27023	0.65259
	GmLac87	13.599	0.02908	0	0	0.009975	2.87219	117.911	0	2.53511
GmLac6-GmLac60	*GmLac6*	1.67197	3.92088	8.33462	0.68082	0.036188	17.817	1.36841	1.64478	0.26018
	GmLac60	1.90439	3.5269	8.78535	0.979661	0.027152	5.6743	0.55415	1.62477	0.29559
GmLac6-GmLac72	*GmLac6*	1.67197	3.92088	8.33462	0.68082	0.036188	17.817	1.36841	1.64478	0.26018
	GmLac72	0.59074	1.08296	1.3223	0.153116	0.056937	10.8463	1.09876	1.00401	0.2863
GmLac70-GmLac71	*GmLac70*	2.01994	5.1544	3.85343	131.307	0.015703	31.122	0.3685	2.14749	1.1624
	GmLac71	0.25852	0.01825	0	1.04685	0	0.90019	11.9166	0	0.20754
GmLac7-GmLac45	*GmLac7*	3.42752	13.1035	18.7042	0.078483	0.152844	0.38836	0.16382	0.0223	0.33882
	GmLac45	0.70011	0.04702	0.03301	6.21115	0.038505	1.63832	16.0232	0	0.82492
GmLac7-GmLac46	*GmLac7*	3.42752	13.1035	18.7042	0.078483	0.152844	0.38836	0.16382	0.0223	0.33882
	GmLac46	3.38403	3.71224	3.92638	71.1852	0.07004	42.1577	0.8744	5.17488	1.1463
GmLac7-GmLac58	*GmLac7*	3.42752	13.1035	18.7042	0.078483	0.152844	0.38836	0.16382	0.0223	0.33882
	GmLac58	0.74271	7.17297	7.1864	6.99751	0.075354	66.2303	1.78565	0.23115	0.69734
GmLac7-GmLac61	*GmLac7*	3.42752	13.1035	18.7042	0.078483	0.152844	0.38836	0.16382	0.0223	0.33882
	GmLac61	1.33023	8.77965	14.2139	0.552605	0.114535	0.90016	0.07608	0.15593	1.45204
GmLac7-GmLac70	*GmLac7*	3.42752	13.1035	18.7042	0.078483	0.152844	0.38836	0.16382	0.0223	0.33882
	GmLac70	2.01994	5.1544	3.85343	131.307	0.015703	31.122	0.3685	2.14749	1.1624
GmLac7-GmLac71	*GmLac7*	3.42752	13.1035	18.7042	0.078483	0.152844	0.38836	0.16382	0.0223	0.33882

	GmLac71	0.25852	0.01825	0	1.04685	0	0.90019	11.9166	0	0.20754
GmLac7-GmLac8	*GmLac7*	3.42752	13.1035	18.7042	0.078483	0.152844	0.38836	0.16382	0.0223	0.33882
	GmLac8	2.95291	4.36308	3.93721	7.97367	0.692242	32.5842	0.56162	0.98136	1.90525
GmLac8-GmLac46	*GmLac8*	2.95291	4.36308	3.93721	7.97367	0.692242	32.5842	0.56162	0.98136	1.90525
	GmLac46	3.38403	3.71224	3.92638	71.1852	0.07004	42.1577	0.8744	5.17488	1.1463
GmLac8-GmLac58	*GmLac8*	2.95291	4.36308	3.93721	7.97367	0.692242	32.5842	0.56162	0.98136	1.90525
	GmLac58	0.74271	7.17297	7.1864	6.99751	0.075354	66.2303	1.78565	0.23115	0.69734
GmLac8-GmLac70	*GmLac8*	2.95291	4.36308	3.93721	7.97367	0.692242	32.5842	0.56162	0.98136	1.90525
	GmLac70	2.01994	5.1544	3.85343	131.307	0.015703	31.122	0.3685	2.14749	1.1624
GmLac9-Glyma.07G146000	*GmLac9*	1.00406	7.28371	15.9315	10.7808	0.614239	75.2652	3.25306	5.61638	2.1843
	Glyma.07G146000	#N/A	#N/A	#N/A	#N/A	#N/A	#N/A	#N/A	#N/A	#N/A
GmLac9-GmLac30	*GmLac9*	1.00406	7.28371	15.9315	10.7808	0.614239	75.2652	3.25306	5.61638	2.1843
	GmLac30	0	0.01036	0	0.006569	0	0.02348	0.0958	0	0.02311
GmLac9-GmLac59	*GmLac9*	1.00406	7.28371	15.9315	10.7808	0.614239	75.2652	3.25306	5.61638	2.1843
	GmLac59	0.43981	3.74448	9.644	15.6223	0.009872	40.7784	1.54436	5.86649	1.01082
GmLac9-GmLac81	*GmLac9*	1.00406	7.28371	15.9315	10.7808	0.614239	75.2652	3.25306	5.61638	2.1843
	GmLac81	0.05663	0.48416	1.08664	8.97794	0	10.1503	0.04955	0.05114	0.22449

Note: Each gene, in a collinear gene pair, expression pattern are list in two adjacent rows from top to bottom. The yellow color block on the right is indicated the expression patterns between the two genes were completely differentiated.

Table S7a. Soybean laccase genes expression pattern following *P. sojae* infection. (Table declares are listed at the bottom of the table)

**Gene id**	**Gene name**	**0 hpi***	**0.5 hpi***	**3 hpi***	**6hpi***	**12 hpi***
Glyma.01G108200	*GmLac1*	0.25129	0.876055	0.314865	0.768322	3.87485
Glyma.01G112600	*GmLac2*	28.6364	21.4187	26.4788	25.1372	20.7291
Glyma.01G173500	*GmLac3*	3.34838	3.12014	3.46683	3.4258	2.48398
Glyma.01G173600	*GmLac4*	12.0184	11.7906	9.01021	7.41335	7.41843
Glyma.01G183100	*GmLac5*	52.014	148.935	38.0585	37.0248	18.0217
Glyma.02G224800	*GmLac6*	13.7993	12.6858	16.5226	15.3783	9.49244
Glyma.02G231600	*GmLac7*	29.1847	30.0099	12.4735	8.59736	5.73199
Glyma.02G261600	*GmLac8*	2.38593	1.98972	2.50418	4.38711	2.66812
Glyma.03G077900	*GmLac9*	29.5307	20.5792	29.441	25.2752	15.7642
Glyma.04G019500	*GmLac10*	22.316	16.8781	23.6979	22.0096	5.41979
Glyma.04G119600	*GmLac11*	5.73385	4.15383	3.66032	3.24543	1.52566
Glyma.05G056100	*GmLac12*	68.893	64.3919	37.0832	44.0194	17.319
Glyma.05G082700	*GmLac13*	0.702849	0.560078	0.59208	0.114705	0.352659
Glyma.06G019800	*GmLac14*	25.8536	29.7824	24.0782	19.2745	6.21724
Glyma.06G284300	*GmLac15*	0.110318	0.109865	0	0	0
Glyma.06G307100	*GmLac16*	30.4785	42.4646	34.2145	24.1045	10.7268
Glyma.06G318800	*GmLac17*	4.79285	3.23413	3.10204	3.73502	2.04709
Glyma.07G054100	*GmLac18*	0.126007	0	0.0631536	0.0642098	0
Glyma.07G054200	*GmLac19*	0.540941	0.0950956	0.127865	0.358264	0.0332556
Glyma.07G133900	*GmLac20*	4.02223	3.12268	3.43984	3.25701	1.48403
Glyma.07G134100	*GmLac21*	4.50357	2.65836	3.06687	3.91382	3.77785
Glyma.07G142400	*GmLac22*	8.13249	7.86233	9.71251	7.74313	5.376
Glyma.07G142500	*GmLac23*	0	0.478863	0.240997	0	0.125642
Glyma.07G142600	*GmLac24*	27.4851	20.842	16.8913	21.9461	21.8717

Glyma.07G225300	*GmLac25*	0.123725	0	0	0.126113	0.194014
Glyma.07G225400	*GmLac26*	0	0	0	0	0
Glyma.07G261800	*GmLac27*	24.6374	11.7348	6.25899	8.06931	3.43014
Glyma.08G138900	*GmLac28*	0	0	0	0.0944281	1.26478
Glyma.08G343500	*GmLac29*	7.12392	5.33623	7.02962	7.03382	4.5278
Glyma.08G353500	*GmLac30*	0	0	0	0	0
Glyma.08G359100	*GmLac31*	10.6903	8.41241	6.80911	4.47284	3.0172
Glyma.08G359200	*GmLac32*	0.321519	0.0800582	0.0804722	0.245168	0
Glyma.08G359300	*GmLac33*	4.23499	3.07961	2.04062	1.87564	0.796766
Glyma.08G359400	*GmLac34*	0	0	0	0	0
Glyma.09G132400	*GmLac35*	0	0	0	0	0
Glyma.10G197300	*GmLac36*	0	0.067923	0	0	0
Glyma.10G219100	*GmLac37*	0	0	0.0624724	0	0
Glyma.10G219200	*GmLac38*	0	0	0	0	0
Glyma.10G263800	*GmLac39*	1.25577	1.13715	1.03033	0.989759	0.477358
Glyma.11G059200	*GmLac40*	52.2743	141.092	36.0018	35.2119	18.506
Glyma.11G069500	*GmLac41*	8.14801	8.17327	6.27185	6.4962	4.2588
Glyma.11G069600	*GmLac42*	10.2873	8.68894	10.7361	9.29235	5.157
Glyma.11G097300	*GmLac43*	5.28157	80.1014	13.6874	5.70817	6.38688
Glyma.11G137500	*GmLac44*	2.05062	2.26955	2.14206	1.74302	1.66727
Glyma.11G164000	*GmLac45*	0.0606944	0	0	0	0
Glyma.11G233400	*GmLac46*	5.18374	4.88284	2.9383	3.27616	2.59161
Glyma.11G236800	*GmLac47*	0	0.0618643	0	0.0633054	0
Glyma.11G256700	*GmLac48*	0.0659301	0	0	0	0
Glyma.12G023300	*GmLac49*	5.04251	89.8709	13.052	3.98345	3.51406
Glyma.12G060900	*GmLac50*	2.34678	1.83206	1.54402	1.65203	0.623523

Glyma.12G097700	*GmLac51*	10.5178	12.6154	8.69565	7.90337	3.8069
Glyma.12G121700	*GmLac52*	2.71466	2.46365	2.47972	1.84511	1.19822
Glyma.12G192800	*GmLac53*	16.4115	10.1854	9.54012	9.23773	6.62683
Glyma.13G033600	*GmLac54*	0	0	0	0	0
Glyma.13G076900	*GmLac55*	117.097	821.016	382.457	147.277	252.838
Glyma.13G338100	*GmLac56*	0	0	0	0	0
Glyma.14G041300	*GmLac57*	0	0.0577009	0.34852	0	0.0605557
Glyma.14G056100	*GmLac58*	4.38833	4.14409	2.85757	3.74943	2.32348
Glyma.14G062300	*GmLac59*	6.48866	4.75081	9.90043	10.1843	5.50746
Glyma.14G191500	*GmLac60*	14.0939	12.4314	12.7878	11.6099	8.54723
Glyma.14G198900	*GmLac61*	21.0053	18.4099	6.76567	4.84649	2.39212
Glyma.14G223000	*GmLac62*	43.5643	28.1977	21.742	31.6227	8.76477
Glyma.15G109000	*GmLac63*	0	0	0	0	0
Glyma.16G158400	*GmLac64*	1.92559	0.516368	0.741796	0.979732	1.31635
Glyma.17G012300	*GmLac65*	96.5633	40.2426	29.4442	45.4458	15.1087
Glyma.17G138300	*GmLac66*	71.2975	61.4042	35.1519	39.8103	18.1958
Glyma.17G180400	*GmLac67*	38.169	35.1837	24.2418	31.2514	14.583
Glyma.17G180500	*GmLac68*	123.461	103.313	37.6063	31.1206	12.1757
Glyma.17G261500	*GmLac69*	102.478	74.2013	58.792	60.5567	21.9033
Glyma.18G023600	*GmLac70*	7.00827	4.845	5.56127	9.1281	5.13901
Glyma.18G057200	*GmLac71*	0.0553463	0	0.111016	0	0.0578558
Glyma.18G065100	*GmLac72*	2.61536	2.6051	2.56265	1.15155	0.932061
Glyma.18G177200	*GmLac73*	4.57646	3.45137	2.22777	2.35348	1.0594
Glyma.18G177300	*GmLac74*	4.93331	3.8716	2.30822	2.24571	1.21898
Glyma.18G177400	*GmLac75*	0.301963	0.120311	0.302738	0.0615709	0
Glyma.18G183500	*GmLac76*	0.0572601	0.057036	0	0.0584027	0

Glyma.18G183700	*GmLac77*	2.14331	1.20432	0.992141	1.85035	1.32131
Glyma.18G193200	*GmLac78*	0.0661296	0.263474	0.198817	0	0
Glyma.18G193300	*GmLac79*	0	0.500434	0.647115	0.073061	0.075041
Glyma.18G193400	*GmLac80*	40.1431	23.0192	84.5537	98.3622	85.4
Glyma.18G197400	*GmLac81*	0.556563	0.221755	0.446543	0.454236	0.23272
Glyma.20G025200	*GmLac82*	0	0	0	0	0
Glyma.20G051600	*GmLac83*	0.677811	0.289311	0.0968291	0.294676	0.405037
Glyma.20G051700	*GmLac84*	6.52082	104.172	13.8237	20.7698	64.6281
Glyma.20G051900	*GmLac85*	79.5271	203.215	104.422	60.3023	18.4677
Glyma.20G126500	*GmLac86*	7.79613	7.56936	4.75486	5.6498	2.39497
Glyma.20G172600	*GmLac87*	0	0	0	0	0
Glyma.20G172700	*GmLac88*	0	0	0	0	0
Glyma.20G192800	*GmLac89*	0.165452	0	0	0.168799	0.115302
Glyma.20G192900	*GmLac90*	0.115672	0	0	0	0
Glyma.U027300	*GmLac91*	0.0560847	1.7877	6.35578	23.0569	73.7541
Glyma.U027400	*GmLac92*	0.791223	6.30493	73.16	310.855	234.526
Glyma.U040600	*GmLac93*	5.53021	74.0722	24.0678	15.1182	50.1238

Note: **hpi** (hours post infection); **FPKM** (Fragments per Kilobase Million); **0 hpi**, **0.5 hpi**, **3 hpi**, **6 hpi**, **12 hpi** indicate gene FPKM value at different stages of infection.

Table S7b. Change value of laccase gene inoculation with P.*sojae*（△FPKM). (Table descriptions are listed at the bottom of the table)

Gene id	Gene name	$\triangle FPKM_{0.5\ hpi}$*	$\triangle FPKM_{3\ hpi}$*	$\triangle FPKM_{6\ hpi}$*	$\triangle FPKM_{12\ hpi}$*
Glyma.01G108200	*GmLac1*	1.797584293	0.324225062	1.608504542	3.941357809
Glyma.01G112600	*GmLac2*	-0.418962242	-0.11300829	-0.188019165	-0.46617344

Glyma.01G173500	*GmLac3*	-0.101820993	0.050139112	0.032967929	-0.430659824
Glyma.01G173600	*GmLac4*	-0.027605389	-0.415572134	-0.696972754	-0.695984619
Glyma.01G183100	*GmLac5*	1.517692886	-0.450671117	-0.490396818	-1.529112566
Glyma.02G224800	*GmLac6*	-0.121371406	0.259828412	0.15629021	-0.539696774
Glyma.02G231600	*GmLac7*	0.040224914	-1.22627968	-1.763128251	-2.347902003
Glyma.02G261600	*GmLac8*	-0.261865942	0.069758008	0.878443433	0.161207911
Glyma.03G077900	*GmLac9*	-0.521007405	-0.004388731	-0.224484822	-0.905520936
Glyma.04G019500	*GmLac10*	-0.402905122	0.086676994	-0.01994463	-2.04156808
Glyma.04G119600	*GmLac11*	-0.464966303	-0.647391881	-0.82090165	-1.909376954
Glyma.05G056100	*GmLac12*	-0.097476719	-0.893573697	-0.646206089	-1.991939414
Glyma.05G082700	*GmLac13*	-0.327064608	-0.247039232	-2.604814745	-0.992906978
Glyma.06G019800	*GmLac14*	0.204087473	-0.102633524	-0.423652701	-2.05584278
Glyma.06G284300	*GmLac15*	-0.005882914	-6.798543084	-6.798543084	-6.798543084
Glyma.06G307100	*GmLac16*	0.478455398	0.166810794	-0.338476882	-1.50648499
Glyma.06G318800	*GmLac17*	-0.567361081	-0.627502473	-0.359682589	-1.226905654
Glyma.07G054100	*GmLac18*	-6.988764203	-0.985305883	-0.961747313	-6.988764203
Glyma.07G054200	*GmLac19*	-2.495593517	-2.072275319	-0.593091419	-3.983724037
Glyma.07G133900	*GmLac20*	-0.36510755	-0.225593408	-0.304363184	-1.43786214
Glyma.07G134100	*GmLac21*	-0.760310306	-0.554152045	-0.20244342	-0.253442142
Glyma.07G142400	*GmLac22*	-0.04873413	0.25611818	-0.07077136	-0.59707306
Glyma.07G142500	*GmLac23*	8.906478768	7.918845353	0	6.984612134
Glyma.07G142600	*GmLac24*	-0.399139273	-0.702336443	-0.324671897	-0.329570898
Glyma.07G225300	*GmLac25*	-6.962606859	-6.962606859	0.027360915	0.644827029
Glyma.07G225400	*GmLac26*	0	0	0	0
Glyma.07G261800	*GmLac27*	-1.069992381	-1.976676314	-1.610212574	-2.844148673
Glyma.08G138900	*GmLac28*	0	0	6.576342243	10.30581096

Glyma.08G343500	*GmLac29*	-0.416782632	-0.019221895	-0.018360306	-0.653744987
Glyma.08G353500	*GmLac30*	0	0	0	0
Glyma.08G359100	*GmLac31*	-0.345674731	-0.65068728	-1.25685172	-1.824676973
Glyma.08G359200	*GmLac32*	-1.99235411	-1.985004383	-0.38974092	-8.333240344
Glyma.08G359300	*GmLac33*	-0.459483132	-1.052984816	-1.174547262	-2.40866164
Glyma.08G359400	*GmLac34*	0	0	0	0
Glyma.09G132400	*GmLac35*	0	0	0	0
Glyma.10G197300	*GmLac36*	6.106913594	0	0	0
Glyma.10G219100	*GmLac37*	0	5.988057489	0	0
Glyma.10G219200	*GmLac38*	0	0	0	0
Glyma.10G263800	*GmLac39*	-0.143029941	-0.285214615	-0.343114575	-1.393558017
Glyma.11G059200	*GmLac40*	1.432445072	-0.538020324	-0.570025389	-1.498058384
Glyma.11G069500	*GmLac41*	0.004465096	-0.377503737	-0.326806685	-0.935839104
Glyma.11G069600	*GmLac42*	-0.243586495	0.06159977	-0.146733969	-0.996120938
Glyma.11G097300	*GmLac43*	3.922533584	1.373641939	0.112041042	0.274097232
Glyma.11G137500	*GmLac44*	0.146278266	0.062908702	-0.234346956	-0.298410741
Glyma.11G164000	*GmLac45*	-5.947067637	-5.947067637	-5.947067637	-5.947067637
Glyma.11G233400	*GmLac46*	-0.086255707	-0.818799028	-0.661825533	-0.999866442
Glyma.11G236800	*GmLac47*	5.974169053	0	6.006867987	0
Glyma.11G256700	*GmLac48*	-6.064583265	-6.064583265	-6.064583265	-6.064583265
Glyma.12G023300	*GmLac49*	4.15537008	1.3718814	-0.340047525	-0.520878803
Glyma.12G060900	*GmLac50*	-0.357043217	-0.603671713	-0.506184319	-1.910470617
Glyma.12G097700	*GmLac51*	0.262330177	-0.274438451	-0.412247744	-1.465902632
Glyma.12G121700	*GmLac52*	-0.139920079	-0.130543959	-0.556814348	-1.179206517
Glyma.12G192800	*GmLac53*	-0.688150736	-0.782564477	-0.82902856	-1.308186507
Glyma.13G033600	*GmLac54*	0	0	0	0

Glyma.13G076900	*GmLac55*	2.80969566	1.70758489	0.330825505	1.110502579
Glyma.13G338100	*GmLac56*	0	0	0	0
Glyma.14G041300	*GmLac57*	5.875310718	8.449231201	0	5.94382055
Glyma.14G056100	*GmLac58*	-0.082597313	-0.61870712	-0.226944724	-0.917092727
Glyma.14G062300	*GmLac59*	-0.449665744	0.609494004	0.650273673	-0.236493855
Glyma.14G191500	*GmLac60*	-0.181068415	-0.140292343	-0.279693438	-0.721475611
Glyma.14G198900	*GmLac61*	-0.190261918	-1.634304134	-2.115512244	-3.133857426
Glyma.14G223000	*GmLac62*	-0.627550824	-1.002628467	-0.462173304	-2.313226747
Glyma.15G109000	*GmLac63*	0	0	0	0
Glyma.16G158400	*GmLac64*	-1.896786854	-1.375011631	-0.974118726	-0.548410884
Glyma.17G012300	*GmLac65*	-1.262730553	-1.713457448	-1.087311206	-2.676014904
Glyma.17G138300	*GmLac66*	-0.215510892	-1.020228013	-0.840693743	-1.970186958
Glyma.17G180400	*GmLac67*	-0.117490975	-0.654882849	-0.288472202	-1.38805272
Glyma.17G180500	*GmLac68*	-0.257031301	-1.71498243	-1.988078929	-3.34187196
Glyma.17G261500	*GmLac69*	-0.465792492	-0.801612002	-0.758945985	-2.226042287
Glyma.18G023600	*GmLac70*	-0.532469794	-0.333590422	0.381208439	-0.447493032
Glyma.18G057200	*GmLac71*	-5.816248973	0.991312033	-5.816248973	0.062863716
Glyma.18G065100	*GmLac72*	-0.00566862	-0.02936175	-1.182731724	-1.487517756
Glyma.18G177200	*GmLac73*	-0.406960189	-1.038299535	-0.959138811	-2.109938704
Glyma.18G177300	*GmLac74*	-0.349545863	-1.095442731	-1.135034439	-2.01599086
Glyma.18G177400	*GmLac75*	-1.320431238	0.0036858	-2.275577852	-8.242997802
Glyma.18G183500	*GmLac76*	-0.005560089	-5.864436273	0.028020329	-5.864436273
Glyma.18G183700	*GmLac77*	-0.831097271	-1.110443027	-0.211935825	-0.69745305
Glyma.18G193200	*GmLac78*	1.978104949	1.573658379	-6.068877141	-6.068877141
Glyma.18G193300	*GmLac79*	8.969916012	9.340106014	6.210642124	6.2487056
Glyma.18G193400	*GmLac80*	-0.802287602	1.07469698	1.292930701	1.089064993

Glyma.18G197400	*GmLac81*	-1.323677012	-0.317108523	-0.292520177	-1.254353632
Glyma.20G025200	*GmLac82*	0	0	0	0
Glyma.20G051600	*GmLac83*	-1.225410706	-2.794674369	-1.198992799	-0.741398745
Glyma.20G051700	*GmLac84*	3.997562968	1.083901645	1.671210255	3.308837372
Glyma.20G051900	*GmLac85*	1.353477387	0.392902903	-0.399227751	-2.106382385
Glyma.20G126500	*GmLac86*	-0.042581281	-0.713236942	-0.46448806	-1.702333344
Glyma.20G172600	*GmLac87*	0	0	0	0
Glyma.20G172700	*GmLac88*	0	0	0	0
Glyma.20G192800	*GmLac89*	-7.378962395	-7.378962395	0.028721757	-0.517230299
Glyma.20G192900	*GmLac90*	-6.866314561	-6.866314561	-6.866314561	-6.866314561
Glyma.U027300	*GmLac91*	4.969663507	6.799048227	8.657941288	10.33542297
Glyma.U027400	*GmLac92*	2.992730664	6.529024398	8.616124124	8.209631721
Glyma.U040600	*GmLac93*	3.743284642	2.121497193	1.450714782	3.179857553

Note: △**FPKM**$_{\mathbf{n\ hpi}}$ were counted according (FPKM$_{n_hpi}$+0.001)/(FPKM$_{0_hpi}$+0.001)

Table S8. Primers used for qRT-PCR.

Gene name	Gene ID	Fprimer	Rprimer
GmLac15	*Glyma.06G284300*	AGCCTCAGTGTCAACCCTTG	TCGGTGAAAACACCCTTGATCT
GmLac18	*Glyma.07G054100*	ACCACAAACACAGTGATGATTG	TGCTACACCCATGGAGTATTTT
GmLac23	*Glyma.07G142500*	ATCATTTAGTTTCCCCGCCTAA	GAAGCTAGAAGGGCAAAAACAA
GmLac25	*Glyma.07G225300*	TCCATCATGAGATTGAGACGAG	GAAAGGGTTGTACATCAGTGAC
GmLac28	*Glyma.08G138900*	TCAACACGCAGAACAACATAAG	TTCTCTTTAAGGGCAATGAGGT
GmLac32	*Glyma.08G359200*	GGGCCCTTCTATAACGGCTC	ATAGCTCGTCGTTGAGTGCC
GmLac36	*Glyma.10G197300*	CATTCCTTGTCACAATAGCCAC	TCAGTAGACACGGGCTTTAAAT
GmLac37	*Glyma.10G219100*	GCAAGAAACTGTTCCTCCAAAT	TTTTGTCGAACAGAGTCTCGTA
GmLac45	*Glyma.11G164000*	CTTCAAGGATTTGCCAAACAGA	ACCTAACAAACATACCGCAAAC
GmLac47	*Glyma.11G236800*	AGACTGGCTTCAAGTCTAAACA	TCCTCACTGAAAACTCCTGAAA
GmLac48	*Glyma.11G256700*	GATGTGGAACCTAAGATCGGAT	TCTGGCATATTGTACTCGTCTC
GmLac57	*Glyma.14G041300*	CCACACATGTGTTTGTCCTTAG	AACAAGGCATGACTACCTTACA
GmLac71	*Glyma.18G057200*	AACAGATCAAGACCAGTTTTGC	AACCCCAATCCCACTATGAAAT
GmLac75	*Glyma.18G177400*	TTCTCTCTCCTTCAAATGGCCC	TTACTTTCCACACTTTCACCTCA
GmLac76	*Glyma.18G183500*	TACACCTTTAACGTCGAGTACC	CCAACGATGGTGAAGTTGTATG
GmLac78	*Glyma.18G193200*	AATGTTAACACGGCTTTCAGAG	AGAACAACTGGAAGAAAAAGCG
GmLac79	*Glyma.18G193300*	CACCAGTTATGTTCGACTTCAC	AATTGAACCTGAGTTTCTTCGC
GmLac89	*Glyma.20G192800*	GACGGTCCACCCTTTGCTAA	GGCCAAAGAATGACACTGCA
GmLac90	*Glyma.20G192900*	ACGATTTGGATGATCCCTTGCT	ATTCCTGCCTGGTTGGATGG
GmLac91	*Glyma.U027300*	AATGAGTCATTTGTGTTGCCTC	TTTGGGTCGGTATAGTCAAACA
GmLac92	*Glyma.U027400*	TTTTTCTAGCATGTTCCTTGGC	GATCACTTGTTGACGACACAAA

Table S9a -first half. Sliding window analysis of nucleotide diversity of 93 laccase gene sequence. (Table descriptions are listed at the bottom of the table)

**Chr**	**Windows**	***G.Soja*$_{SNP}$***	**PI$_{soja}$***	***G.Max*$_{SNP}$***	**PI$_{Max}$***	**$\pi_{Max/Soja}$***	**Gene id**	**Gene name**
Chr01	36930001-36950000	730	0.00937337	398	0.000528105	0.056340996	*Glyma.01G108200*	*GmLac1*
Chr01	36940001-36960000	693	0.00658368	464	0.000834595	0.126767249	*Glyma.01G108200*	*GmLac1*
Chr01	36950001-36970000	614	0.00418733	453	0.00104559	0.249703272	*Glyma.01G108200*	*GmLac1*
Chr01	38350001-38370000	566	0.00527249	360	0.00269323	0.510807986	*Glyma.01G112600*	*GmLac2*
Chr01	38340001-38360000	624	0.00596893	416	0.00235611	0.394729039	*Glyma.01G112600*	*GmLac2*
Chr01	51030001-51050000	192	0.00143632	112	0.00089814	0.625306338	*Glyma.01G173500*	*GmLac3*
Chr01	51040001-51060000	291	0.00217039	150	0.00151536	0.698197098	*Glyma.01G173500*	*GmLac3*
Chr01	51040001-51060000	291	0.00217039	150	0.00151536	0.698197098	*Glyma.01G173600*	*GmLac4*
Chr01	51050001-51070000	313	0.00247773	161	0.00146324	0.590556679	*Glyma.01G173600*	*GmLac4*
Chr01	51830001-51850000	237	0.00226797	155	0.00161155	0.710569364	*Glyma.01G183100*	*GmLac5*
Chr01	51840001-51860000	307	0.00287032	186	0.00205184	0.714847125	*Glyma.01G183100*	*GmLac5*
Chr02	41210001-41230000	702	0.00623179	478	0.00363794	0.583771276	*Glyma.02G224800*	*GmLac6*
Chr02	41220001-41240000	526	0.00569231	354	0.00385108	0.676540807	*Glyma.02G224800*	*GmLac6*
Chr02	41880001-41900000	332	0.00329296	233	0.000661599	0.20091316	*Glyma.02G231600*	*GmLac7*
Chr02	41870001-41890000	450	0.00443845	279	0.00109056	0.245707398	*Glyma.02G231600*	*GmLac7*
Chr02	44740001-44760000	278	0.00226741	120	0.000280899	0.123885402	*Glyma.02G261600*	*GmLac8*
Chr02	44750001-44770000	395	0.00385523	217	0.000843029	0.218671519	*Glyma.02G261600*	*GmLac8*
Chr02	44760001-44780000	377	0.00379832	239	0.000964546	0.253940163	*Glyma.02G261600*	*GmLac8*
Chr03	19350001-19370000	547	0.00430366	388	0.00300067	0.697236771	*Glyma.03G077900*	*GmLac9*
Chr03	19360001-19380000	279	0.00257038	239	0.00167205	0.650506929	*Glyma.03G077900*	*GmLac9*
Chr04	1510001-1530000	366	0.00346365	160	0.000843645	0.243571088	*Glyma.04G019500*	*GmLac10*
Chr04	1520001-1540000	296	0.0024632	125	0.00073875	0.299914745	*Glyma.04G019500*	*GmLac10*
Chr04	1530001-1550000	359	0.00287238	185	0.00148012	0.515293937	*Glyma.04G019500*	*GmLac10*

Chr04	14430001-14450000	502	0.00357429	364	0.00280357	0.784371162	*Glyma.04G119600*	*GmLac11*
Chr04	14440001-14460000	455	0.0030526	384	0.00245702	0.804894189	*Glyma.04G119600*	*GmLac11*
Chr05	5120001-5140000	529	0.00538196	302	0.00253696	0.471382173	*Glyma.05G056100*	*GmLac12*
Chr05	5110001-5130000	495	0.0035485	175	0.000395522	0.111461744	*Glyma.05G056100*	*GmLac12*
Chr05	13140001-13160000	478	0.00291806	307	0.000715739	0.245279055	*Glyma.05G082700*	*GmLac13*
Chr05	13150001-13170000	387	0.00268302	223	0.000906046	0.337696327	*Glyma.05G082700*	*GmLac13*
Chr05	13160001-13180000	390	0.00243363	232	0.000857239	0.352247055	*Glyma.05G082700*	*GmLac13*
Chr06	1480001-1500000	260	0.00282996	176	0.00248023	0.876418748	*Glyma.06G019800*	*GmLac14*
Chr06	1490001-1510000	310	0.00338772	182	0.00268794	0.793436293	*Glyma.06G019800*	*GmLac14*
Chr06	1500001-1520000	493	0.00396918	234	0.00192551	0.485115313	*Glyma.06G019800*	*GmLac14*
Chr06	47260001-47280000	611	0.00701206	484	0.00494092	0.704631734	*Glyma.06G284300*	*GmLac15*
Chr06	47270001-47290000	488	0.00580068	381	0.00346475	0.597300661	*Glyma.06G284300*	*GmLac15*
Chr06	49580001-49600000	427	0.00386868	271	0.00264424	0.683499281	*Glyma.06G307100*	*GmLac16*
Chr06	49590001-49610000	440	0.00424575	292	0.00278352	0.655601484	*Glyma.06G307100*	*GmLac16*
Chr06	50730001-50750000	355	0.00310356	268	0.00216819	0.69861385	*Glyma.06G318800*	*GmLac17*
Chr06	50740001-50760000	301	0.00270298	190	0.001311	0.485020237	*Glyma.06G318800*	*GmLac17*
Chr07	4720001-4740000	510	0.0038874	347	0.00136823	0.351965324	*Glyma.07G054100*	*GmLac18*
Chr07	4730001-4750000	614	0.00566682	439	0.00230477	0.406713112	*Glyma.07G054100*	*GmLac18*
Chr07	4730001-4750000	614	0.00566682	439	0.00230477	0.406713112	*Glyma.07G054200*	*GmLac19*
Chr07	4740001-4760000	436	0.00465591	317	0.00213253	0.458026465	*Glyma.07G054200*	*GmLac19*
Chr07	15850001-15870000	518	0.00498313	273	0.00334753	0.671772561	*Glyma.07G133900*	*GmLac20*
Chr07	15860001-15880000	438	0.00455026	242	0.00310937	0.683338974	*Glyma.07G133900*	*GmLac20*
Chr07	15870001-15890000	390	0.00390222	267	0.00291414	0.746790289	*Glyma.07G133900*	*GmLac20*
Chr07	15870001-15890000	390	0.00390222	267	0.00291414	0.746790289	*Glyma.07G134100*	*GmLac21*
Chr07	15880001-15900000	967	0.00961172	705	0.00672423	0.699586546	*Glyma.07G134100*	*GmLac21*
Chr07	16910001-16930000	279	0.00213618	149	0.000867887	0.406279902	*Glyma.07G142400*	*GmLac22*

Chr07	16920001-16940000	254	0.00226783	144	0.00103746	0.457468152	*Glyma.07G142400*	*GmLac22*
Chr07	16930001-16950000	339	0.00498756	242	0.00399746	0.801486097	*Glyma.07G142500*	*GmLac23*
Chr07	16910001-16930000	279	0.00213618	149	0.000867887	0.406279902	*Glyma.07G142500*	*GmLac23*
Chr07	16920001-16940000	254	0.00226783	144	0.00103746	0.457468152	*Glyma.07G142500*	*GmLac23*
Chr07	16960001-16980000	865	0.0100486	521	0.00588316	0.585470613	*Glyma.07G142600*	*GmLac24*
Chr07	16970001-16990000	566	0.00572423	333	0.00353453	0.617468201	*Glyma.07G142600*	*GmLac24*
Chr07	16980001-17000000	601	0.0045201	358	0.000637917	0.141128957	*Glyma.07G142600*	*GmLac24*
Chr07	40230001-40250000	470	0.00386678	295	0.000500863	0.129529738	*Glyma.07G225300*	*GmLac25*
Chr07	40220001-40240000	492	0.00421592	324	0.000593821	0.140852056	*Glyma.07G225300*	*GmLac25*
Chr07	40240001-40260000	459	0.00426001	241	0.000766763	0.179990892	*Glyma.07G225300*	*GmLac25*
Chr07	40230001-40250000	470	0.00386678	295	0.000500863	0.129529738	*Glyma.07G225400*	*GmLac26*
Chr07	40250001-40270000	488	0.00485898	271	0.00083755	0.172371568	*Glyma.07G225400*	*GmLac26*
Chr07	40240001-40260000	459	0.00426001	241	0.000766763	0.179990892	*Glyma.07G225400*	*GmLac26*
Chr07	43670001-43690000	271	0.00269008	154	0.00134068	0.49837923	*Glyma.07G261800*	*GmLac27*
Chr07	43660001-43680000	305	0.0027403	156	0.00057741	0.210710506	*Glyma.07G261800*	*GmLac27*
Chr08	10620001-10640000	307	0.00344297	196	0.00235465	0.683900818	*Glyma.08G138900*	*GmLac28*
Chr08	10630001-10650000	270	0.00319221	185	0.00216181	0.677214218	*Glyma.08G138900*	*GmLac28*
Chr08	45860001-45880000	276	0.00243106	138	0.000398728	0.164014051	*Glyma.08G343500*	*GmLac29*
Chr08	45870001-45890000	372	0.0029697	171	0.000863614	0.290808499	*Glyma.08G343500*	*GmLac29*
Chr08	46650001-46670000	343	0.00277655	191	0.00148836	0.536046533	*Glyma.08G353500*	*GmLac30*
Chr08	46660001-46680000	256	0.0019618	148	0.0012119	0.617749006	*Glyma.08G353500*	*GmLac30*
Chr08	47090001-47110000	378	0.0040851	250	0.00232146	0.56827495	*Glyma.08G359100*	*GmLac31*
Chr08	47100001-47120000	333	0.00289398	188	0.00104228	0.360154528	*Glyma.08G359100*	*GmLac31*
Chr08	47110001-47130000	314	0.00270613	167	0.000849501	0.313917291	*Glyma.08G359200*	*GmLac32*
Chr08	47100001-47120000	333	0.00289398	188	0.00104228	0.360154528	*Glyma.08G359200*	*GmLac32*
Chr08	47110001-47130000	314	0.00270613	167	0.000849501	0.313917291	*Glyma.08G359300*	*GmLac33*

Chr08	47100001-47120000	333	0.00289398	188	0.00104228	0.360154528	*Glyma.08G359300*	*GmLac33*
Chr08	47120001-47140000	339	0.00336336	201	0.0013645	0.405695495	*Glyma.08G359300*	*GmLac33*
Chr08	47110001-47130000	314	0.00270613	167	0.000849501	0.313917291	*Glyma.08G359400*	*GmLac34*
Chr08	47120001-47140000	339	0.00336336	201	0.0013645	0.405695495	*Glyma.08G359400*	*GmLac34*
Chr09	33010001-33030000	503	0.00509609	376	0.00176684	0.346705023	*Glyma.09G132400*	*GmLac35*
Chr09	33000001-33020000	595	0.00428404	426	0.00187464	0.437586951	*Glyma.09G132400*	*GmLac35*
Chr10	42850001-42870000	254	0.00180153	144	0.00107813	0.598452427	*Glyma.10G197300*	*GmLac36*
Chr10	42860001-42880000	339	0.00321741	188	0.00183069	0.568994937	*Glyma.10G197300*	*GmLac36*
Chr10	42840001-42860000	446	0.00429963	267	0.00137244	0.319199559	*Glyma.10G197300*	*GmLac36*
Chr10	45090001-45110000	705	0.00695382	486	0.00215421	0.309788001	*Glyma.10G219100*	*GmLac37*
Chr10	45080001-45100000	750	0.00747836	528	0.00246158	0.329160404	*Glyma.10G219100*	*GmLac37*
Chr10	45100001-45120000	287	0.00264907	165	0.00127537	0.481440657	*Glyma.10G219100*	*GmLac37*
Chr10	45090001-45110000	705	0.00695382	486	0.00215421	0.309788001	*Glyma.10G219200*	*GmLac38*
Chr10	45100001-45120000	287	0.00264907	165	0.00127537	0.481440657	*Glyma.10G219200*	*GmLac38*
Chr10	48790001-48810000	323	0.00305026	220	0.00179265	0.587703999	*Glyma.10G263800*	*GmLac39*
Chr10	48800001-48820000	338	0.00279549	213	0.00153821	0.550247005	*Glyma.10G263800*	*GmLac39*
Chr10	48810001-48830000	343	0.00258343	178	0.00131576	0.509307394	*Glyma.10G263800*	*GmLac39*
Chr11	4460001-4480000	145	0.00131895	119	0.000702842	0.532879942	*Glyma.11G059200*	*GmLac40*
Chr11	4470001-4490000	135	0.00104405	110	0.000547756	0.524645371	*Glyma.11G059200*	*GmLac40*
Chr11	5230001-5250000	250	0.00251455	157	0.00134672	0.535570977	*Glyma.11G069500*	*GmLac41*
Chr11	5220001-5240000	270	0.00279037	169	0.00107878	0.386608228	*Glyma.11G069500*	*GmLac41*
Chr11	5230001-5250000	250	0.00251455	157	0.00134672	0.535570977	*Glyma.11G069600*	*GmLac42*
Chr11	5240001-5260000	347	0.00331467	229	0.00217974	0.657603924	*Glyma.11G069600*	*GmLac42*
Chr11	5220001-5240000	270	0.00279037	169	0.00107878	0.386608228	*Glyma.11G069600*	*GmLac42*
Chr11	7410001-7430000	549	0.00510391	354	0.00265393	0.51997978	*Glyma.11G097300*	*GmLac43*
Chr11	7420001-7440000	465	0.00417178	280	0.00229188	0.549377005	*Glyma.11G097300*	*GmLac43*

Chr11	7430001-7450000	289	0.00183421	156	0.00111708	0.609025139	*Glyma.11G097300*	*GmLac43*
Chr11	10440001-10460000	283	0.00222294	140	0.000516469	0.232336005	*Glyma.11G137500*	*GmLac44*
Chr11	10450001-10470000	355	0.00325268	222	0.000949166	0.291810446	*Glyma.11G137500*	*GmLac44*
Chr11	10460001-10480000	431	0.00355691	293	0.00126857	0.356649451	*Glyma.11G137500*	*GmLac44*
Chr11	15440001-15460000	741	0.00500975	608	0.00235974	0.471029492	*Glyma.11G164000*	*GmLac45*
Chr11	15430001-15450000	378	0.00287292	256	0.000932625	0.324626164	*Glyma.11G164000*	*GmLac45*
Chr11	32870001-32890000	409	0.00435149	200	0.00096279	0.221255248	*Glyma.11G233400*	*GmLac46*
Chr11	32860001-32880000	372	0.00425862	148	0.00104086	0.244412509	*Glyma.11G233400*	*GmLac46*
Chr11	33140001-33160000	354	0.00330338	238	0.00225278	0.681962112	*Glyma.11G236800*	*GmLac47*
Chr11	33150001-33170000	364	0.00330163	221	0.00301466	0.913082326	*Glyma.11G236800*	*GmLac47*
Chr11	33160001-33180000	349	0.00355064	227	0.00267211	0.752571367	*Glyma.11G236800*	*GmLac47*
Chr11	34620001-34640000	359	0.00252409	235	0.00184832	0.732271829	*Glyma.11G256700*	*GmLac48*
Chr11	34630001-34650000	292	0.00200657	183	0.00136604	0.680783626	*Glyma.11G256700*	*GmLac48*
Chr11	34640001-34660000	188	0.00110141	105	0.000870653	0.790489464	*Glyma.11G256700*	*GmLac48*
Chr12	1710001-1730000	445	0.00485299	342	0.0015344	0.316176213	*Glyma.12G023300*	*GmLac49*
Chr12	1700001-1720000	493	0.00494105	354	0.00162522	0.32892199	*Glyma.12G023300*	*GmLac49*
Chr12	4410001-4430000	449	0.00551803	322	0.000902936	0.163633761	*Glyma.12G060900*	*GmLac50*
Chr12	4420001-4440000	410	0.00380713	287	0.0007855	0.206323398	*Glyma.12G060900*	*GmLac50*
Chr12	8390001-8410000	355	0.0033349	229	0.000780431	0.234019311	*Glyma.12G097700*	*GmLac51*
Chr12	8380001-8400000	441	0.00432698	285	0.00132986	0.307341379	*Glyma.12G097700*	*GmLac51*
Chr12	13080001-13100000	874	0.00803301	554	0.000428107	0.053293473	*Glyma.12G121700*	*GmLac52*
Chr12	13090001-13110000	525	0.00426974	296	0.000352186	0.082484179	*Glyma.12G121700*	*GmLac52*
Chr12	35430001-35450000	632	0.00649206	409	0.00109204	0.168211631	*Glyma.12G192800*	*GmLac53*
Chr12	35420001-35440000	350	0.00291273	262	0.00135502	0.465206181	*Glyma.12G192800*	*GmLac53*
Chr13	10560001-10580000	1848	0.0124698	1507	0.0106516	0.854191727	*Glyma.13G033600*	*GmLac54*
Chr13	10570001-10590000	1157	0.00684365	936	0.00591123	0.863753991	*Glyma.13G033600*	*GmLac54*

Chr13	18180001-18200000	263	0.00190207	192	0.00222595	1.170277645	*Glyma.13G076900*	*GmLac55*
Chr13	18190001-18210000	357	0.00265	249	0.00274509	1.035883019	*Glyma.13G076900*	*GmLac55*
Chr13	18200001-18220000	408	0.0028123	251	0.00231536	0.823297657	*Glyma.13G076900*	*GmLac55*
Chr13	43080001-43100000	379	0.00328336	160	8.81E-05	0.026819021	*Glyma.13G338100*	*GmLac56*
Chr13	43090001-43110000	451	0.00408431	204	0.000243096	0.059519478	*Glyma.13G338100*	*GmLac56*
Chr13	43070001-43090000	479	0.00487904	228	0.000303264	0.06215649	*Glyma.13G338100*	*GmLac56*
Chr14	3100001-3120000	555	0.00491879	416	0.00338755	0.688695797	*Glyma.14G041300*	*GmLac57*
Chr14	3110001-3130000	524	0.00378197	353	0.00277474	0.733675836	*Glyma.14G041300*	*GmLac57*
Chr14	3120001-3140000	516	0.003948	368	0.00295316	0.748014184	*Glyma.14G041300*	*GmLac57*
Chr14	4440001-4460000	561	0.0049094	289	0.00250333	0.509905487	*Glyma.14G056100*	*GmLac58*
Chr14	4450001-4470000	302	0.00319218	176	0.00165413	0.518181932	*Glyma.14G056100*	*GmLac58*
Chr14	5060001-5080000	332	0.00321653	148	0.00121231	0.376899951	*Glyma.14G062300*	*GmLac59*
Chr14	5070001-5090000	291	0.00275641	148	0.00110385	0.400466549	*Glyma.14G062300*	*GmLac59*
Chr14	45600001-45620000	810	0.00750269	436	0.00255954	0.341149641	*Glyma.14G191500*	*GmLac60*
Chr14	45610001-45630000	493	0.00614782	327	0.00320318	0.521026966	*Glyma.14G191500*	*GmLac60*
Chr14	45620001-45640000	331	0.00411562	204	0.00171467	0.416624956	*Glyma.14G191500*	*GmLac60*
Chr14	46390001-46410000	287	0.00271051	191	0.001736	0.640469875	*Glyma.14G198900*	*GmLac61*
Chr14	46400001-46420000	305	0.00258147	210	0.00177316	0.686879956	*Glyma.14G198900*	*GmLac61*
Chr14	48800001-48820000	351	0.00259067	157	0.000640116	0.247085117	*Glyma.14G223000*	*GmLac62*
Chr14	48810001-48830000	399	0.00343652	228	0.00120776	0.351448558	*Glyma.14G223000*	*GmLac62*
Chr15	8550001-8570000	236	0.00160515	137	0.00135349	0.843217145	*Glyma.15G109000*	*GmLac63*
Chr15	8560001-8580000	320	0.00212594	202	0.00170451	0.801767689	*Glyma.15G109000*	*GmLac63*
Chr16	31860001-31880000	425	0.00409214	357	0.00289503	0.707461133	*Glyma.16G158400*	*GmLac64*
Chr16	31850001-31870000	548	0.00510888	409	0.00309077	0.604979956	*Glyma.16G158400*	*GmLac64*
Chr17	940001-960000	313	0.00311104	212	0.000907682	0.291761597	*Glyma.17G012300*	*GmLac65*
Chr17	950001-970000	422	0.00324871	228	0.000965743	0.297269686	*Glyma.17G012300*	*GmLac65*

Chr17	11170001-11190000	442	0.0040206	227	0.000447539	0.111311496	*Glyma.17G138300*	*GmLac66*
Chr17	11180001-11200000	615	0.00580905	373	0.00260572	0.448562157	*Glyma.17G138300*	*GmLac66*
Chr17	20470001-20490000	405	0.0019947	244	0.00154802	0.776066576	*Glyma.17G180400*	*GmLac67*
Chr17	20480001-20500000	308	0.00144083	172	0.00125822	0.873260551	*Glyma.17G180400*	*GmLac67*
Chr17	20490001-20510000	207	0.00107763	106	0.000941892	0.874040255	*Glyma.17G180400*	*GmLac67*
Chr17	20560001-20580000	389	0.00214242	254	0.00241208	1.12586701	*Glyma.17G180500*	*GmLac68*
Chr17	20570001-20590000	247	0.00146068	129	0.00139393	0.954302106	*Glyma.17G180500*	*GmLac68*
Chr17	20580001-20600000	270	0.00164753	160	0.00146461	0.88897319	*Glyma.17G180500*	*GmLac68*
Chr17	41530001-41550000	239	0.00206636	101	0.000372189	0.180118179	*Glyma.17G261500*	*GmLac69*
Chr17	41520001-41540000	254	0.00190624	99	0.000424839	0.22286753	*Glyma.17G261500*	*GmLac69*
Chr17	41510001-41530000	247	0.00132044	87	0.000539798	0.408801612	*Glyma.17G261500*	*GmLac69*
Chr18	1710001-1730000	264	0.00266594	212	0.00174438	0.654320802	*Glyma.18G023600*	*GmLac70*
Chr18	1720001-1740000	346	0.00322403	263	0.00234196	0.726407633	*Glyma.18G023600*	*GmLac70*
Chr18	5030001-5050000	613	0.00412119	354	0.00310039	0.752304553	*Glyma.18G057200*	*GmLac71*
Chr18	5040001-5060000	268	0.00212393	153	0.00168963	0.795520568	*Glyma.18G057200*	*GmLac71*
Chr18	5910001-5930000	280	0.00272068	191	0.000441346	0.162219004	*Glyma.18G065100*	*GmLac72*
Chr18	5920001-5940000	669	0.00605375	468	0.0011853	0.195795994	*Glyma.18G065100*	*GmLac72*
Chr18	5930001-5950000	538	0.00459581	381	0.00107244	0.233351683	*Glyma.18G065100*	*GmLac72*
Chr18	42230001-42250000	556	0.00736779	462	0.00633248	0.859481608	*Glyma.18G177200*	*GmLac73*
Chr18	42240001-42260000	216	0.00287478	188	0.00258714	0.899943648	*Glyma.18G177200*	*GmLac73*
Chr18	42240001-42260000	216	0.00287478	188	0.00258714	0.899943648	*Glyma.18G177300*	*GmLac74*
Chr18	42250001-42270000	564	0.00722949	502	0.00770216	1.065380822	*Glyma.18G177300*	*GmLac74*
Chr18	42310001-42330000	310	0.00347424	247	0.00304669	0.876937114	*Glyma.18G177400*	*GmLac75*
Chr18	42320001-42340000	677	0.005518	569	0.00439041	0.79565241	*Glyma.18G177400*	*GmLac75*
Chr18	44250001-44270000	435	0.00491061	330	0.00125107	0.254768756	*Glyma.18G183500*	*GmLac76*
Chr18	44260001-44280000	377	0.00378138	261	0.00106101	0.280588039	*Glyma.18G183500*	*GmLac76*

Chr18	44260001-44280000	377	0.00378138	261	0.00106101	0.280588039	*Glyma.18G183700*	*GmLac77*
Chr18	44270001-44290000	496	0.00372776	505	0.00214457	0.575297229	*Glyma.18G183700*	*GmLac77*
Chr18	46540001-46560000	878	0.00853125	687	0.00211755	0.248210989	*Glyma.18G193200*	*GmLac78*
Chr18	46550001-46570000	932	0.00884977	724	0.00254434	0.287503517	*Glyma.18G193200*	*GmLac78*
Chr18	46560001-46580000	774	0.00745971	622	0.0021778	0.291941644	*Glyma.18G193200*	*GmLac78*
Chr18	46570001-46590000	440	0.00411183	354	0.0011781	0.286514763	*Glyma.18G193300*	*GmLac79*
Chr18	46560001-46580000	774	0.00745971	622	0.0021778	0.291941644	*Glyma.18G193300*	*GmLac79*
Chr18	46580001-46600000	391	0.00258966	267	0.00100892	0.389595545	*Glyma.18G193300*	*GmLac79*
Chr18	46610001-46630000	949	0.00618184	671	0.00184881	0.29907115	*Glyma.18G193400*	*GmLac80*
Chr18	46620001-46640000	937	0.00866634	738	0.00275146	0.317488121	*Glyma.18G193400*	*GmLac80*
Chr18	47370001-47390000	946	0.00774244	688	0.0035964	0.46450473	*Glyma.18G197400*	*GmLac81*
Chr18	47360001-47380000	819	0.0058289	598	0.00294412	0.505090154	*Glyma.18G197400*	*GmLac81*
Chr20	2740001-2760000	472	0.00372635	316	0.00132037	0.354333329	*Glyma.20G025200*	*GmLac82*
Chr20	2730001-2750000	498	0.00414326	338	0.00155725	0.375851383	*Glyma.20G025200*	*GmLac82*
Chr20	11610001-11630000	954	0.00649227	594	0.000986528	0.151954247	*Glyma.20G051600*	*GmLac83*
Chr20	11620001-11640000	1077	0.00775181	716	0.00204658	0.264013179	*Glyma.20G051600*	*GmLac83*
Chr20	11680001-11700000	444	0.00396309	248	0.000137896	0.034795072	*Glyma.20G051700*	*GmLac84*
Chr20	11690001-11710000	287	0.00201797	155	0.000269759	0.1336784	*Glyma.20G051700*	*GmLac84*
Chr20	11670001-11690000	972	0.0100248	626	0.00140258	0.139911021	*Glyma.20G051700*	*GmLac84*
Chr20	11750001-11770000	786	0.00525711	394	0.000586574	0.111577273	*Glyma.20G051900*	*GmLac85*
Chr20	11760001-11780000	480	0.00297301	240	0.000383626	0.129036229	*Glyma.20G051900*	*GmLac85*
Chr20	11770001-11790000	370	0.00230645	160	0.000663046	0.287474691	*Glyma.20G051900*	*GmLac85*
Chr20	36850001-36870000	560	0.00564491	419	0.00359228	0.636375071	*Glyma.20G126500*	*GmLac86*
Chr20	36860001-36880000	573	0.00534109	405	0.00347716	0.651020672	*Glyma.20G126500*	*GmLac86*
Chr20	41000001-41020000	324	0.00331727	254	0.0011142	0.335878599	*Glyma.20G172600*	*GmLac87*
Chr20	41010001-41030000	317	0.00222603	228	0.00115539	0.519036132	*Glyma.20G172600*	*GmLac87*

Chr20	41020001-41040000	426	0.0029295	256	0.00134993	0.460805598	*Glyma.20G172600*	*GmLac87*
Chr20	41010001-41030000	317	0.00222603	228	0.00115539	0.519036132	*Glyma.20G172700*	*GmLac88*
Chr20	41020001-41040000	426	0.0029295	256	0.00134993	0.460805598	*Glyma.20G172700*	*GmLac88*
Chr20	43170001-43190000	309	0.0035503	223	0.00278231	0.783683069	*Glyma.20G192800*	*GmLac89*
Chr20	43180001-43200000	348	0.00374143	223	0.00270431	0.722801175	*Glyma.20G192800*	*GmLac89*
Chr20	43190001-43210000	351	0.00368865	223	0.00169007	0.458181177	*Glyma.20G192800*	*GmLac89*
Chr20	43180001-43200000	348	0.00374143	223	0.00270431	0.722801175	*Glyma.20G192900*	*GmLac90*
Chr20	43190001-43210000	351	0.00368865	223	0.00169007	0.458181177	*Glyma.20G192900*	*GmLac90*

Table S9a - latter half. Sliding window analysis of nucleotide diversity of 93 laccase gene sequence. (Table descriptions are listed at the bottom of the table)

**Gene id**	**Gene name**	**Physical location***	**Overlap***	**chr_Mean ($\pi_{Max/Soja}$)***	**△π_{Ratio}***
Glyma.01G108200	*GmLac1*	36947944-36957036	2056	0.460792348	-0.404451352
Glyma.01G108200	*GmLac1*	36947944-36957036	9092	0.460792348	-0.334025099
Glyma.01G108200	*GmLac1*	36947944-36957036	7035	0.460792348	-0.211089076
Glyma.01G112600	*GmLac2*	38353594-38358163	4569	0.460792348	0.050015638
Glyma.01G112600	*GmLac2*	38353594-38358163	4569	0.460792348	-0.066063309
Glyma.01G173500	*GmLac3*	51045827-51048552	2725	0.460792348	0.16451399
Glyma.01G173500	*GmLac3*	51045827-51048552	2725	0.460792348	0.23740475
Glyma.01G173600	*GmLac4*	51051683-51054726	3043	0.460792348	0.23740475
Glyma.01G173600	*GmLac4*	51051683-51054726	3043	0.460792348	0.129764331
Glyma.01G183100	*GmLac5*	51843424-51847358	3934	0.460792348	0.249777016
Glyma.01G183100	*GmLac5*	51843424-51847358	3934	0.460792348	0.254054777
Glyma.02G224800	*GmLac6*	41222747-41227060	4313	0.507494768	0.076276508
Glyma.02G224800	*GmLac6*	41222747-41227060	4313	0.507494768	0.169046039
Glyma.02G231600	*GmLac7*	41882853-41885826	2973	0.507494768	-0.306581608

Glyma.02G231600	*GmLac7*	41882853-41885826	2973	0.507494768	-0.26178737
Glyma.02G261600	*GmLac8*	44758969-44763198	1031	0.507494768	-0.383609366
Glyma.02G261600	*GmLac8*	44758969-44763198	4229	0.507494768	-0.288823249
Glyma.02G261600	*GmLac8*	44758969-44763198	3197	0.507494768	-0.253554605
Glyma.03G077900	*GmLac9*	19361933-19366381	4448	0.594576536	0.102660235
Glyma.03G077900	*GmLac9*	19361933-19366381	4448	0.594576536	0.055930393
Glyma.04G019500	*GmLac10*	1525860-1533999	4140	0.543613008	-0.30004192
Glyma.04G019500	*GmLac10*	1525860-1533999	8139	0.543613008	-0.243698263
Glyma.04G019500	*GmLac10*	1525860-1533999	3998	0.543613008	-0.028319071
Glyma.04G119600	*GmLac11*	14443110-14447983	4873	0.543613008	0.240758154
Glyma.04G119600	*GmLac11*	14443110-14447983	4873	0.543613008	0.261281181
Glyma.05G056100	*GmLac12*	5123859-5128473	4614	0.378758439	0.092623734
Glyma.05G056100	*GmLac12*	5123859-5128473	4614	0.378758439	-0.267296695
Glyma.05G082700	*GmLac13*	13157352-13160970	2648	0.378758439	-0.133479384
Glyma.05G082700	*GmLac13*	13157352-13160970	3618	0.378758439	-0.041062112
Glyma.05G082700	*GmLac13*	13157352-13160970	969	0.378758439	-0.026511384
Glyma.06G019800	*GmLac14*	1494230-1501925	5770	0.567000154	0.309418594
Glyma.06G019800	*GmLac14*	1494230-1501925	7695	0.567000154	0.226436139
Glyma.06G019800	*GmLac14*	1494230-1501925	1924	0.567000154	-0.081884841
Glyma.06G284300	*GmLac15*	47271762-47274605	2843	0.567000154	0.13763158
Glyma.06G284300	*GmLac15*	47271762-47274605	2843	0.567000154	0.030300507
Glyma.06G307100	*GmLac16*	49594181-49599176	4995	0.567000154	0.116499127
Glyma.06G307100	*GmLac16*	49594181-49599176	4995	0.567000154	0.08860133
Glyma.06G318800	*GmLac17*	50742178-50747062	4884	0.567000154	0.131613696
Glyma.06G318800	*GmLac17*	50742178-50747062	4884	0.567000154	-0.081979917
Glyma.07G054100	*GmLac18*	4733502-4736103	2601	0.468204013	-0.116238689

Glyma.07G054100	*GmLac18*	4733502-4736103	2601	0.468204013	-0.061490901
Glyma.07G054200	*GmLac19*	4742597-4747000	4403	0.468204013	-0.061490901
Glyma.07G054200	*GmLac19*	4742597-4747000	4403	0.468204013	-0.010177548
Glyma.07G133900	*GmLac20*	15868910-15873228	1090	0.468204013	0.203568548
Glyma.07G133900	*GmLac20*	15868910-15873228	4318	0.468204013	0.215134961
Glyma.07G133900	*GmLac20*	15868910-15873228	3227	0.468204013	0.278586276
Glyma.07G134100	*GmLac21*	15880168-15883379	3211	0.468204013	0.278586276
Glyma.07G134100	*GmLac21*	15880168-15883379	3211	0.468204013	0.231382533
Glyma.07G142400	*GmLac22*	16921847-16926142	4295	0.468204013	-0.061924111
Glyma.07G142400	*GmLac22*	16921847-16926142	4295	0.468204013	-0.010735861
Glyma.07G142500	*GmLac23*	16929178-16933812	3811	0.468204013	0.333282084
Glyma.07G142500	*GmLac23*	16929178-16933812	822	0.468204013	-0.061924111
Glyma.07G142500	*GmLac23*	16929178-16933812	4634	0.468204013	-0.010735861
Glyma.07G142600	*GmLac24*	16975838-16981175	4162	0.468204013	0.1172666
Glyma.07G142600	*GmLac24*	16975838-16981175	5337	0.468204013	0.149264188
Glyma.07G142600	*GmLac24*	16975838-16981175	1174	0.468204013	-0.327075056
Glyma.07G225300	*GmLac25*	40239363-40241517	2154	0.468204013	-0.338674275
Glyma.07G225300	*GmLac25*	40239363-40241517	637	0.468204013	-0.327351957
Glyma.07G225300	*GmLac25*	40239363-40241517	1516	0.468204013	-0.288213121
Glyma.07G225400	*GmLac26*	40248750-40251212	1250	0.468204013	-0.338674275
Glyma.07G225400	*GmLac26*	40248750-40251212	1211	0.468204013	-0.295832445
Glyma.07G225400	*GmLac26*	40248750-40251212	2462	0.468204013	-0.288213121
Glyma.07G261800	*GmLac27*	43671355-43676147	4792	0.468204013	0.030175217
Glyma.07G261800	*GmLac27*	43671355-43676147	4792	0.468204013	-0.257493507
Glyma.08G138900	*GmLac28*	10636329-10638034	1705	0.431595212	0.252305606
Glyma.08G138900	*GmLac28*	10636329-10638034	1705	0.431595212	0.245619006

Glyma.08G343500	*GmLac29*	45872298-45879050	6752	0.431595212	-0.267581161
Glyma.08G343500	*GmLac29*	45872298-45879050	6752	0.431595212	-0.140786713
Glyma.08G353500	*GmLac30*	46665729-46668560	2831	0.431595212	0.104451321
Glyma.08G353500	*GmLac30*	46665729-46668560	2831	0.431595212	0.186153794
Glyma.08G359100	*GmLac31*	47105388-47108727	3339	0.431595212	0.136679738
Glyma.08G359100	*GmLac31*	47105388-47108727	3339	0.431595212	-0.071440684
Glyma.08G359200	*GmLac32*	47112555-47115278	2723	0.431595212	-0.117677921
Glyma.08G359200	*GmLac32*	47112555-47115278	2723	0.431595212	-0.071440684
Glyma.08G359300	*GmLac33*	47117261-47120794	3533	0.431595212	-0.117677921
Glyma.08G359300	*GmLac33*	47117261-47120794	2739	0.431595212	-0.071440684
Glyma.08G359300	*GmLac33*	47117261-47120794	793	0.431595212	-0.025899717
Glyma.08G359400	*GmLac34*	47124368-47126962	2594	0.431595212	-0.117677921
Glyma.08G359400	*GmLac34*	47124368-47126962	2594	0.431595212	-0.025899717
Glyma.09G132400	*GmLac35*	33011543-33014363	2820	0.549423281	-0.202718258
Glyma.09G132400	*GmLac35*	33011543-33014363	2820	0.549423281	-0.11183633
Glyma.10G197300	*GmLac36*	42857995-42860990	2995	0.520235126	0.078217301
Glyma.10G197300	*GmLac36*	42857995-42860990	989	0.520235126	0.048759811
Glyma.10G197300	*GmLac36*	42857995-42860990	2005	0.520235126	-0.201035567
Glyma.10G219100	*GmLac37*	45096674-45100122	3448	0.520235126	-0.210447125
Glyma.10G219100	*GmLac37*	45096674-45100122	3326	0.520235126	-0.191074722
Glyma.10G219100	*GmLac37*	45096674-45100122	121	0.520235126	-0.038794469
Glyma.10G219200	*GmLac38*	45102350-45106419	4069	0.520235126	-0.210447125
Glyma.10G219200	*GmLac38*	45102350-45106419	4069	0.520235126	-0.038794469
Glyma.10G263800	*GmLac39*	48805999-48811533	4001	0.520235126	0.067468873
Glyma.10G263800	*GmLac39*	48805999-48811533	5534	0.520235126	0.030011879
Glyma.10G263800	*GmLac39*	48805999-48811533	1532	0.520235126	-0.010927732

Glyma.11G059200	*GmLac40*	4474609-4478620	4011	0.391354363	0.141525579
Glyma.11G059200	*GmLac40*	4474609-4478620	4011	0.391354363	0.133291008
Glyma.11G069500	*GmLac41*	5231970-5235047	3077	0.391354363	0.144216614
Glyma.11G069500	*GmLac41*	5231970-5235047	3077	0.391354363	-0.004746135
Glyma.11G069600	*GmLac42*	5237575-5240089	2514	0.391354363	0.144216614
Glyma.11G069600	*GmLac42*	5237575-5240089	88	0.391354363	0.266249561
Glyma.11G069600	*GmLac42*	5237575-5240089	2425	0.391354363	-0.004746135
Glyma.11G097300	*GmLac43*	7426344-7431229	3656	0.391354363	0.128625417
Glyma.11G097300	*GmLac43*	7426344-7431229	4885	0.391354363	0.158022642
Glyma.11G097300	*GmLac43*	7426344-7431229	1228	0.391354363	0.217670776
Glyma.11G137500	*GmLac44*	10456742-10461383	3258	0.391354363	-0.159018358
Glyma.11G137500	*GmLac44*	10456742-10461383	4641	0.391354363	-0.099543917
Glyma.11G137500	*GmLac44*	10456742-10461383	1382	0.391354363	-0.034704912
Glyma.11G164000	*GmLac45*	15441595-15444592	2997	0.391354363	0.079675129
Glyma.11G164000	*GmLac45*	15441595-15444592	2997	0.391354363	-0.066728199
Glyma.11G233400	*GmLac46*	32873226-32877927	4701	0.391354363	-0.170099115
Glyma.11G233400	*GmLac46*	32873226-32877927	4701	0.391354363	-0.146941854
Glyma.11G236800	*GmLac47*	33157016-33161441	2984	0.391354363	0.290607749
Glyma.11G236800	*GmLac47*	33157016-33161441	4425	0.391354363	0.521727963
Glyma.11G236800	*GmLac47*	33157016-33161441	1440	0.391354363	0.361217004
Glyma.11G256700	*GmLac48*	34639433-34641547	567	0.391354363	0.340917466
Glyma.11G256700	*GmLac48*	34639433-34641547	2114	0.391354363	0.289429263
Glyma.11G256700	*GmLac48*	34639433-34641547	1546	0.391354363	0.399135101
Glyma.12G023300	*GmLac49*	1711347-1715917	4570	0.381038111	-0.064861898
Glyma.12G023300	*GmLac49*	1711347-1715917	4570	0.381038111	-0.052116121
Glyma.12G060900	*GmLac50*	4423650-4427688	4038	0.381038111	-0.21740435

Glyma.12G060900	*GmLac50*	4423650-4427688	4038	0.381038111	-0.174714713
Glyma.12G097700	*GmLac51*	8390539-8395322	4783	0.381038111	-0.1470188
Glyma.12G097700	*GmLac51*	8390539-8395322	4783	0.381038111	-0.073696732
Glyma.12G121700	*GmLac52*	13091668-13095659	3991	0.381038111	-0.327744638
Glyma.12G121700	*GmLac52*	13091668-13095659	3991	0.381038111	-0.298553932
Glyma.12G192800	*GmLac53*	35434955-35439253	4298	0.381038111	-0.21282648
Glyma.12G192800	*GmLac53*	35434955-35439253	4298	0.381038111	0.08416807
Glyma.13G033600	*GmLac54*	10575983-10578863	2880	0.560028083	0.294163644
Glyma.13G033600	*GmLac54*	10575983-10578863	2880	0.560028083	0.303725908
Glyma.13G076900	*GmLac55*	18197033-18205367	2967	0.560028083	0.610249562
Glyma.13G076900	*GmLac55*	18197033-18205367	8334	0.560028083	0.475854936
Glyma.13G076900	*GmLac55*	18197033-18205367	5366	0.560028083	0.263269574
Glyma.13G338100	*GmLac56*	43086954-43090229	3275	0.560028083	-0.533209062
Glyma.13G338100	*GmLac56*	43086954-43090229	228	0.560028083	-0.500508605
Glyma.13G338100	*GmLac56*	43086954-43090229	3046	0.560028083	-0.497871593
Glyma.14G041300	*GmLac57*	3116248-3122893	3752	0.460699875	0.227995922
Glyma.14G041300	*GmLac57*	3116248-3122893	6645	0.460699875	0.272975961
Glyma.14G041300	*GmLac57*	3116248-3122893	2892	0.460699875	0.287314309
Glyma.14G056100	*GmLac58*	4452088-4456161	4073	0.460699875	0.049205612
Glyma.14G056100	*GmLac58*	4452088-4456161	4073	0.460699875	0.057482057
Glyma.14G062300	*GmLac59*	5075491-5079469	3978	0.460699875	-0.083799924
Glyma.14G062300	*GmLac59*	5075491-5079469	3978	0.460699875	-0.060233326
Glyma.14G191500	*GmLac60*	45616466-45620640	3534	0.460699875	-0.119550234
Glyma.14G191500	*GmLac60*	45616466-45620640	4174	0.460699875	0.060327091
Glyma.14G191500	*GmLac60*	45616466-45620640	639	0.460699875	-0.044074919
Glyma.14G198900	*GmLac61*	46404283-46407292	3009	0.460699875	0.17977

Glyma.14G198900	*GmLac61*	46404283-46407292	3009	0.460699875	0.226180081
Glyma.14G223000	*GmLac62*	48811743-48817333	5590	0.460699875	-0.213614758
Glyma.14G223000	*GmLac62*	48811743-48817333	5590	0.460699875	-0.109251317
Glyma.15G109000	*GmLac63*	8563266-8566506	3240	0.651790365	0.19142678
Glyma.15G109000	*GmLac63*	8563266-8566506	3240	0.651790365	0.149977324
Glyma.16G158400	*GmLac64*	31863760-31867327	3567	0.635639222	0.071821911
Glyma.16G158400	*GmLac64*	31863760-31867327	3567	0.635639222	-0.030659266
Glyma.17G012300	*GmLac65*	950397-955232	4835	0.626645128	-0.334883531
Glyma.17G012300	*GmLac65*	950397-955232	4835	0.626645128	-0.329375442
Glyma.17G138300	*GmLac66*	11183728-11188961	5233	0.626645128	-0.515333632
Glyma.17G138300	*GmLac66*	11183728-11188961	5233	0.626645128	-0.178082971
Glyma.17G180400	*GmLac67*	20487778-20491962	2222	0.626645128	0.149421448
Glyma.17G180400	*GmLac67*	20487778-20491962	4184	0.626645128	0.246615423
Glyma.17G180400	*GmLac67*	20487778-20491962	1961	0.626645128	0.247395127
Glyma.17G180500	*GmLac68*	20576340-20582231	3660	0.626645128	0.499221882
Glyma.17G180500	*GmLac68*	20576340-20582231	5891	0.626645128	0.327656978
Glyma.17G180500	*GmLac68*	20576340-20582231	2230	0.626645128	0.262328062
Glyma.17G261500	*GmLac69*	41526584-41534998	4997	0.626645128	-0.446526949
Glyma.17G261500	*GmLac69*	41526584-41534998	8414	0.626645128	-0.403777598
Glyma.17G261500	*GmLac69*	41526584-41534998	3416	0.626645128	-0.217843516
Glyma.18G023600	*GmLac70*	1723409-1728464	5055	0.644706686	0.009614116
Glyma.18G023600	*GmLac70*	1723409-1728464	5055	0.644706686	0.081700947
Glyma.18G057200	*GmLac71*	5045036-5048066	3030	0.644706686	0.107597867
Glyma.18G057200	*GmLac71*	5045036-5048066	3030	0.644706686	0.150813882
Glyma.18G065100	*GmLac72*	5925631-5930200	4369	0.644706686	-0.482487682
Glyma.18G065100	*GmLac72*	5925631-5930200	4569	0.644706686	-0.448910692

Glyma.18G065100	*GmLac72*	5925631-5930200	199	0.644706686	-0.411355003
Glyma.18G177200	*GmLac73*	42244982-42247802	2820	0.644706686	0.214774922
Glyma.18G177200	*GmLac73*	42244982-42247802	2820	0.644706686	0.255236962
Glyma.18G177300	*GmLac74*	42252244-42255072	2828	0.644706686	0.255236962
Glyma.18G177300	*GmLac74*	42252244-42255072	2828	0.644706686	0.420674136
Glyma.18G177400	*GmLac75*	42324003-42327195	3192	0.644706686	0.232230428
Glyma.18G177400	*GmLac75*	42324003-42327195	3192	0.644706686	0.150945724
Glyma.18G183500	*GmLac76*	44265156-44269294	4138	0.644706686	-0.38993793
Glyma.18G183500	*GmLac76*	44265156-44269294	4138	0.644706686	-0.364118647
Glyma.18G183700	*GmLac77*	44276577-44279879	3302	0.644706686	-0.364118647
Glyma.18G183700	*GmLac77*	44276577-44279879	3302	0.644706686	-0.069409457
Glyma.18G193200	*GmLac78*	46559987-46565879	13	0.644706686	-0.396495697
Glyma.18G193200	*GmLac78*	46559987-46565879	5892	0.644706686	-0.357203169
Glyma.18G193200	*GmLac78*	46559987-46565879	5878	0.644706686	-0.352765042
Glyma.18G193300	*GmLac79*	46576771-46581234	4463	0.644706686	-0.358191923
Glyma.18G193300	*GmLac79*	46576771-46581234	3229	0.644706686	-0.352765042
Glyma.18G193300	*GmLac79*	46576771-46581234	1233	0.644706686	-0.255111141
Glyma.18G193400	*GmLac80*	46622868-46628926	6058	0.644706686	-0.345635536
Glyma.18G193400	*GmLac80*	46622868-46628926	6058	0.644706686	-0.327218565
Glyma.18G197400	*GmLac81*	47370198-47374339	4141	0.644706686	-0.180201956
Glyma.18G197400	*GmLac81*	47370198-47374339	4141	0.644706686	-0.139616532
Glyma.20G025200	*GmLac82*	2743092-2745282	2190	0.442490108	-0.088156779
Glyma.20G025200	*GmLac82*	2743092-2745282	2190	0.442490108	-0.066638725
Glyma.20G051600	*GmLac83*	11622159-11626958	4799	0.442490108	-0.290535861
Glyma.20G051600	*GmLac83*	11622159-11626958	4799	0.442490108	-0.178476929
Glyma.20G051700	*GmLac84*	11685747-11690052	4305	0.442490108	-0.407695036

Glyma.20G051700	*GmLac84*	11685747-11690052	51	0.442490108	-0.308811708
Glyma.20G051700	*GmLac84*	11685747-11690052	4253	0.442490108	-0.302579087
Glyma.20G051900	*GmLac85*	11769051-11777145	949	0.442490108	-0.330912835
Glyma.20G051900	*GmLac85*	11769051-11777145	8094	0.442490108	-0.313453879
Glyma.20G051900	*GmLac85*	11769051-11777145	7144	0.442490108	-0.155015417
Glyma.20G126500	*GmLac86*	36862931-36869680	6749	0.442490108	0.193884963
Glyma.20G126500	*GmLac86*	36862931-36869680	6749	0.442490108	0.208530564
Glyma.20G172600	*GmLac87*	41019363-41023633	637	0.442490108	-0.106611509
Glyma.20G172600	*GmLac87*	41019363-41023633	4270	0.442490108	0.076546024
Glyma.20G172600	*GmLac87*	41019363-41023633	3632	0.442490108	0.01831549
Glyma.20G172700	*GmLac88*	41025526-41028921	3395	0.442490108	0.076546024
Glyma.20G172700	*GmLac88*	41025526-41028921	3395	0.442490108	0.01831549
Glyma.20G192800	*GmLac89*	43186683-43190491	3317	0.442490108	0.341192961
Glyma.20G192800	*GmLac89*	43186683-43190491	3808	0.442490108	0.280311067
Glyma.20G192800	*GmLac89*	43186683-43190491	490	0.442490108	0.015691069
Glyma.20G192900	*GmLac90*	43192981-43196211	3230	0.442490108	0.280311067
Glyma.20G192900	*GmLac90*	43192981-43196211	3230	0.442490108	0.015691069

Note: **Windows** indicate a 10kb region on chromosome; ***G.Soja***$_{SNP}$ indicate SNP numbers obatined when comparing corresponding region between *G.soja* species with reference genome; **PI**$_{\mathbf{soja}}$ indicate nucleotide diversity (π) of a specific window in *G.soja* species; ***G.Max***$_{SNP}$ indicate SNP numbers obatined when comparing corresponding region between *G.max* species with reference genome; **PI**$_{\mathbf{Max}}$ indicate nucleotide diversity (π) of a specific window in *G.max* species; $\boldsymbol{\pi}_{Max/Soja}$ = **PI**$_{\mathbf{Max}}$ / **PI**$_{\mathbf{soja}}$**; Physical location** indicate chromosome location of a laccase gene; **Overlap** means overlap sequence length (bp) of a specific window and a laccase gene. **chr_Mean (**$\boldsymbol{\pi}_{Max/Soja}$**)** indicate the average π value of the a entire chromosome; $\triangle\boldsymbol{\pi}_{\mathbf{Ratio}}$ = $\boldsymbol{\pi}_{Max/Soja}$ **- chr_Mean (**$\boldsymbol{\pi}_{Max/Soja}$**)**.

Table S9b. List of selection *GmLacs* above average value. (Table descriptions are listed at the bottom of the table)

GmLac1	GmLac32	GmLac59
GmLac2	GmLac33	GmLac60
GmLac7	GmLac34	GmLac62
GmLac8	GmLac35	GmLac64
GmLac10	GmLac36	GmLac65
GmLac12	GmLac37	GmLac66
GmLac13	GmLac38	GmLac69
GmLac14	GmLac39	GmLac72
GmLac17	GmLac41	GmLac76
GmLac18	GmLac42	GmLac77
GmLac19	GmLac44	GmLac78
GmLac23	GmLac45	GmLac79
GmLac22	GmLac46	GmLac80
GmLac24	GmLac49	GmLac81
GmLac25	GmLac50	GmLac82
GmLac26	GmLac51	GmLac83
GmLac27	GmLac52	GmLac84
GmLac29	GmLac53	GmLac85
GmLac31	GmLac56	GmLac87

The list shows the genes $\triangle\boldsymbol{\pi}_{\mathbf{Ratio}}$ **< 0**

Table S9c. List of GmLacs experience sellection pressure above top 5% genome-wide level.

GmLac1	*Glyma.01G108200*
GmLac8	*Glyma.02G261600*
GmLac12	*Glyma.05G056100*
GmLac25	*Glyma.07G225300*
GmLac26	*Glyma.07G225400*
GmLac52	*Glyma.12G121700*
GmLac56	*Glyma.13G338100*
GmLac66	*Glyma.17G138300*
GmLac84	*Glyma.20G051700*
GmLac85	*Glyma.20G051900*

Table S9d . Expression pattern of the 10 highly selected *GmLacs*.

Gene name	Root	RH	Nodules	Pod	Seed	Stem	SAM	Leaves	Flower	scale(value)
GmLac1	41.64	4.94	6.82	0.06	0.00	0.04	0.01	0.03	0.14	0
GmLac8	2.95	4.36	3.94	7.97	0.69	32.58	0.56	0.98	1.91	5
GmLac12	54.73	65.77	60.52	23.36	22.16	47.63	55.08	6.07	5.52	10
GmLac25	0.20	0.00	0.00	0.01	0.00	1.06	0.20	0.03	505.03	15
GmLac26	0.00	0.00	0.00	0.00	0.00	0.06	0.00	0.00	22.85	20
GmLac52	0.18	0.59	0.63	4.39	0.46	1.15	0.01	0.23	0.10	25
GmLac56	0.00	0.00	0.00	1.37	0.00	0.00	0.21	0.00	0.02	30
GmLac66	66.83	56.05	48.36	56.52	35.88	84.13	43.27	8.40	9.79	
GmLac84	23.66	2.72	2.76	0.15	0.09	1.48	0.06	0.14	0.33	
GmLac85	93.65	67.15	30.28	32.47	44.21	48.51	13.49	12.04	2.55	

Note: Refer to Additional file 6 (sheet 1).

Data S1. SNPs of the ten genes among 302 re-sequenced soybean accessions are publicly available at:
https://figshare.com/s/9066a35ea35bfaba4282
